# Demographic history differences between Hispanics and Brazilians imprint haplotype features

**DOI:** 10.1093/g3journal/jkac111

**Published:** 2022-05-02

**Authors:** Pedro Rodrigues Sousa da Cruz, Galina Ananina, Rodrigo Secolin, Vera Lúcia Gil-da-Silva-Lopes, Carmen Silvia Passos Lima, Paulo Henrique Condeixa de França, Amanda Donatti, Gustavo Jacob Lourenço, Tânia Kawasaki de Araujo, Milena Simioni, Iscia Lopes-Cendes, Fernando Ferreira Costa, Mônica Barbosa de Melo

**Affiliations:** Laboratory of Human Genetics, Center for Molecular Biology and Genetic Engineering (CBMEG), University of Campinas—UNICAMP, Campinas, SP 13083-875, Brazil; Laboratory of Human Genetics, Center for Molecular Biology and Genetic Engineering (CBMEG), University of Campinas—UNICAMP, Campinas, SP 13083-875, Brazil; Department of Medical Genetics and Genomic Medicine, School of Medical Sciences, University of Campinas—UNICAMP, Campinas, SP 13083-887, Brazil; The Brazilian Institute of Neuroscience and Neurotechnology (BRAINN), Campinas, SP 13083-887, Brazil; Department of Medical Genetics and Genomic Medicine, School of Medical Sciences, University of Campinas—UNICAMP, Campinas, SP 13083-887, Brazil; Clinical Oncology Service, Department of Internal Medicine, School of Medical Sciences, University of Campinas—UNICAMP, Campinas, SP 13083-887, Brazil; Joinville Stroke Biobank, University of Region of Joinville—UNIVILLE, Joinville, SC 89202-190, Brazil; Department of Medical Genetics and Genomic Medicine, School of Medical Sciences, University of Campinas—UNICAMP, Campinas, SP 13083-887, Brazil; The Brazilian Institute of Neuroscience and Neurotechnology (BRAINN), Campinas, SP 13083-887, Brazil; Laboratory of Cancer Genetics, School of Medical Sciences, University of Campinas—UNICAMP, Campinas, SP 13083-887, Brazil; Department of Medical Genetics and Genomic Medicine, School of Medical Sciences, University of Campinas—UNICAMP, Campinas, SP 13083-887, Brazil; Department of Medical Genetics and Genomic Medicine, School of Medical Sciences, University of Campinas—UNICAMP, Campinas, SP 13083-887, Brazil; Department of Medical Genetics and Genomic Medicine, School of Medical Sciences, University of Campinas—UNICAMP, Campinas, SP 13083-887, Brazil; The Brazilian Institute of Neuroscience and Neurotechnology (BRAINN), Campinas, SP 13083-887, Brazil; Hematology and Hemotherapy Center, University of Campinas—UNICAMP, Campinas, SP, 13083-878, Brazil; Laboratory of Human Genetics, Center for Molecular Biology and Genetic Engineering (CBMEG), University of Campinas—UNICAMP, Campinas, SP 13083-875, Brazil

**Keywords:** Latinos, haplotypes, population, selection, ROH, IBD sharing, linkage disequilibrium, diversity

## Abstract

Admixture is known to greatly impact the genetic landscape of a population and, while genetic variation underlying human phenotypes has been shown to differ among populations, studies on admixed subjects are still scarce. Latin American populations are the result of complex demographic history, such as 2 or 3-way admixing events, bottlenecks and/or expansions, and adaptive events unique to the American continent. To explore the impact of these events on the genetic structure of Latino populations, we evaluated the following haplotype features: linkage disequilibrium, shared identity by descent segments, runs of homozygosity, and extended haplotype homozygosity (integrated haplotype score) in Latinos represented in the 1000 Genome Project along with array data from 171 Brazilians sampled in the South and Southeast regions of Brazil. We found that linkage disequilibrium decay relates to the amount of American and African ancestry. The extent of identity by descent sharing positively correlates with historical effective population sizes, which we found to be steady or growing, except for Puerto Ricans and Colombians. Long runs of homozygosity, a particular instance of autozygosity, was only enriched in Peruvians and Native Americans. We used simulations to account for random sampling and linkage disequilibrium to filter positive selection indexes and found 244 unique markers under selection, 26 of which are common to 2 or more populations. Some markers exhibiting positive selection signals had estimated time to the most recent common ancestor consistent with human adaptation to the American continent. In conclusion, Latino populations present highly divergent haplotype characteristics that impact genetic architecture and underlie complex phenotypes.

## Introduction

Latin Americans inhabit continental Latin America and the Caribbean and are the largest ethnic minority in the United States (United Nations 2019). Despite being arguably the largest group of admixed populations on the globe, with around 600 million people, the genetic variation of Latin Americans is poorly explored compared to other populations (Adhikari *et al.* 2017). Contemporary Latino populations are formed by a complex blend of many ethnic groups. European colonization had reached virtually the whole of Latin America by the beginning of the 17th century, while the English colonies in North America were restricted to a limited portion of its territory (Bethell 2008). Another distinctive feature of Latin American formation was the extent of mixing between natives and Europeans, which was generally more pervasive as compared to other colonies on the continent (Bethell 2008). Newcomers to Latin America encountered advanced civilizations with sizable populations that perished from warfare, diseases, and slavery (Elliott 2008). Iberian colonial policies created a focus on exploitation that attracted more male conquistadors, resulting in forced and sex-biased relations between white men and Native-American women (Morner 1968; Alves-Silva *et al.* 2000; Elliott 2008; Bryc *et al.* 2010; Conomos *et al.* 2016).

The admixture was extended by 2 incremental pulses of migration: the trans-Atlantic slave trade (from the 16th to the 19th century) and post-Colonial migrations (19th and 20th centuries). A major influx of European, Middle Eastern, Japanese, and Chinese migrants formed the current Latino populations, along with the extant indigenous people and sub-Saharan Africans and/or their descendants (Morner 1968).

These demographic events largely impact haplotype structure (Ruiz-Linares *et al.* 2014; Martin *et al.* 2017, 2018). Consisting of a string of alleles that are physically linked in the same chromosome, haplotypes are statistically more informative than individual unphased genotypes, due to dimensionality reduction (Clark 2004). Therefore, from the methodological standpoint, there is an intrinsic benefit in regarding genetic variation as phased haplotypes instead of a handful of separate SNPs, since statistical power is increased (Clark 2004). Currently, there is an increasing number of statistical methods that can improve accuracy in haplotype phasing (Loh *et al.* 2016) and robustness to phase uncertainty (Seltman *et al.* 2003; Howie *et al.* 2012; Guan 2014; Xu and Guan 2014).

Allelic association studies, the most common strategy for establishing genotype-phenotype links, also rely on haplotype structure, since flanking alleles are usually used as markers for a core causal region. This nonrandom statistical association between alleles, called linkage disequilibrium (LD), therefore, greatly impacts association studies. Admixture events can create LD (admixture LD) between all loci with divergent allele frequencies in genomic regions of different source populations, thus allowing to map genes related to a given trait (Pfaff *et al.* 2001; Tishkoff and Verrelli 2003). Admixture is also expected to increase LD between unlinked alleles, which may cause spurious association signals (Pfaff *et al.* 2001; Rosenberg and Nordborg 2006). Both true signal and false-positive effects depend on the particular admixture process that took place (e.g. hybridization and isolation), which impacts the extent of LD decay of the admixed population (Zhou *et al.* 2017).

Moreover, extended haplotypes inherited from a recent common ancestor, known as identical by descent (IBD), reflect genetic relationships between individuals and can be used to identify fine-scale population structure (Gusev *et al.* 2012; Moreno-Estrada *et al.* 2013; Dai *et al.* 2020) as well as to infer recent demographic history (Browning and Browning 2015; Browning *et al.* 2018). Large haplotypes that are IBD may be present in the same individual and loci of homologous chromosomes, causing runs of homozygosity (ROH). ROHs are important in the efforts to evaluate complex human traits because they raise homozygosity rates in many low-frequency alleles (Ceballos, Joshi, *et al.* 2018). ROHs are also important to improve the understanding of demographics since they carry clues to the level of consanguinity and, likewise, on the timeframe of the admixture event (Ceballos, Joshi, *et al.* 2018). Extended haplotype homozygosity (EHH), when shared by a substantial portion of a population, might also be indicative of events of recent positive selection (Sabeti *et al.* 2002; Hanchard *et al.* 2006), one metric aimed at the detection of selection is the integrated haplotype score (iHS), proposed by Voight *et al.* (2006).

Characterizing these haplotype features (LD, IBD sharing, ROH, and iHS) is necessary to comprehend how admixture impacts the genetic structure of admixed populations as well as to assist in genetic studies’ methodological design. Our study, therefore, aims to expand the characterization of admixed Latin American populations in terms of haplotype structure. In order to accomplish this goal, we (1) quantified LD in Latino samples and compared it with other populations around the world; (2) quantified identity by descent (IBD) segments in the Latin American samples and compared them with reference populations; (3) characterized ancestry components and described the genetic structure in the samples; (4) quantified ROH segment sharing; as well as (5) evaluated EHH (iHS) among admixed Latin American populations. We sought to evaluate populations with different admixture histories and therefore our dataset consists of Hispanic populations of the 1000 Genomes Project Consortium (2015) (Puerto Ricans, Peruvians, Colombians, and Mexicans; Table 1), along with samples from 2 locations in Brazil (BR) sampled from its southern and southeastern regions. The Brazilian regions were elected on account of a major wave of recent migration.

**Table 1.** Labels of populations used in the present study.

Population labels	Description	Super-population code
CEU	Utah residents with Northern and Western European ancestry	EUR
FIN	Finnish in Finland	EUR
GBR	British in England and Scotland	EUR
IBS	Iberian Population in Spain	EUR
TSI	Tuscans in Italy	EUR
ASW	Americans of African Ancestry in SW USA	AFR
ACB	African Caribbeans in Barbados	AFR
ESN	Esan in Nigeria	AFR
GWD	Gambians in Western Division, the Gambia	AFR
LWK	Luhya in Webuye, Kenya	AFR
MSL	Mende in Sierra Leone	AFR
YRI	Yoruba in Ibadan, Nigeria	AFR
BR	Brazilians from São Paulo and Santa Catarina States; Brazil	AMR
CLM	Colombians from Medellin, Colombia	AMR
MXL	Mexican Ancestry from Los Angeles USA	AMR
PEL	Peruvians in Lima, Peru	AMR
PUR	Puerto Ricans from Puerto Rico	AMR
NAM	Native Americans	AMR

Native American samples were obtained from Mao *et al.* (2007), and correspond to 43 samples from Nahua (*n* = 10) and Maya (*n* = 6), Mexico; Quechua (*n* = 2) Peru, and Aymara (*n* = 25), Bolivia.

## Materials and methods

### Ethics statement

This study followed the principles of the Declaration of Helsinki and Brazilian ethical guidelines for biomedical research involving humans. All participants signed an informed consent form. Ethics approval was obtained from the local Research Ethics Committees under the following protocols: CAAE35316314.9.1001.5404, CAAE0413.0.146.000-09, 0241.0.146.000-05, CAAE12112913.3.0000.5404, 25000.142907/2013-07, and 25000.142907/2013-07.

### Brazilian subjects

We analyzed 171 Brazilian individuals, including 4 groups: control cohorts from the projects (1) “Assessment of copy number variations in the susceptibility to stroke in patients with sickle cell anemia” (*N* = 31, noncarriers of the sickle cell mutation), (2) “High-density microarray technique in the assessment of copy number variation in congenital defects of complex inheritance: oral clefts as a model” (*N* = 20) (Simioni *et al.* 2015), (3) the participants of the project “Identification of susceptibility genes for squamous cell carcinoma of the tongue by large scale genotyping” (*N* = 94), and (4) the Joinville Stroke Registry (*N* = 26). Geographically, the sampling process occurred in 2 Brazilian cities: Campinas (1,164,098 inhabitants; São Paulo State, Brazil’s Southeast), and Joinville (562,151 inhabitants; Santa Catarina State, Brazil’s South) (IBGE 2010a).

### Reference populations

We also made use of publicly available data from the 1000 Genomes Project (1000 Genomes Project Consortium 2010), along with 43 samples of Native Americans in our study. The latter population is described in Mao *et al.* (2007). These samples were genotyped using Affymetrix 6.0 platform; and pertain to the following populations: Nahua (*n* = 10) and Maya (*n* = 6), Mexico; Quechua (*n* = 2), Peru; and Aymara (*n* = 25), Bolivia.

### Genotyping

Genomic DNA was extracted from the peripheral blood of each Brazilian participant using the QIAamp DNA Blood Midi Kit (Qiagen, Hilden, Germany) and genotyped by 2 platforms: Affymetrix Genome-Wide SNP Arrays 6.0 and 5.0 (Affymetrix, CA, USA). While preparing the DNA samples, we rigorously followed the manufacturer’s instructions. The files containing scanned images were examined with Genotyping Console software v4.1.3, using the default settings. We applied the following genotyping algorithms: BRLMM (Affymetrix 2006; Rabbee and Speed 2006) for the Affymetrix Genome-Wide SNP Array 5.0 and the Birdseed v2 (Korn *et al.* 2008) algorithm for the SNP Array 6.0. The human genome assembly used for genotyping was GRCh37/hg19. After filtering out A/T and G/C genotypes, the number of successfully genotyped autosomal SNPs in common between the Genome-Wide SNP Array 5.0 and 6.0 platforms was 305,060.

### Quality Control

The raw genotype data underwent quality control (QC) using PLINK software (v1.07 and v1.9) (Purcell *et al.* 2007; Chang *et al.* 2015). Each population was examined at 2 levels: by subject and by marker*. Subject QC*: each sample was checked for discordance concerning sex registration, genotype call rate, and the presence of duplicated or related samples. We also removed known regions of long-range LD in human populations (Price *et al.* 2008). We removed one sample from a pair of second degree based on the IBD coefficient (PI-HAT > 0.1875), and no sample was found to be duplicated (i.e. no pair had PI-HAT > 0.98). *Marker QC*: we removed markers with high missing genotyping rates (>0.05), low minor allele frequency (< 0.05), and those that deviated from the Hardy–Weinberg equilibrium (*P*-value < 1e-6). After going through these filters, one Brazilian sample was removed and the final genotyping rate was 99.94%. *Merging datasets:* only autosomal SNPs were kept in our analysis.

Additionally, we removed SNPs that were either A/T or G/C, since it is not possible to assure concordant location of alleles regarding positive or negative strands. At this point, we kept 196,749 SNPs used for LD decay and FineStructure. For all other analyses, we also pruned LD using a window of 1,000 SNPs moving every 50 SNPs, with an *r*^2^ threshold of 0.5, thus keeping 176,390 markers. Of these, most are intergenic, 166,250, whereas genic markers account for 7,264 (intronic) and 2,876 (exonic) markers. The average marker distance is 14,507 bp (±2,226 bp; see Supplementary Table 1 for distribution along chromosomes).

### Phasing

All samples were simultaneously phased. This procedure was done with the aid of the SHAPEIT program v2 (Delaneau *et al.* 2014; O’Connell *et al.* 2014). Phasing was performed with the following reference panel: “1,000 Genomes haplotypes—Phase I integrated variant set release (SHAPEIT2) in NCBI build 37 (hg19) coordinates.”

### Population structure

To explore population stratification, we applied 2 analytical tools: ADMIXTURE v1.3 (Alexander *et al.* 2009) and FineStructure v2 (Lawson *et al.* 2012). The ADMIXTURE model-based algorithm does not account for LD information explicitly, as such the removal of linked SNPs is recommended to reduce background LD (Alexander *et al.* 2009; Lawson *et al.* 2012). To identify the optimal value of *K*, we ran ADMIXTURE 10 times for each *K* from 2 to 12, using different random seeds for each run. We compared cross-validation errors (CVEs) averaged across the 10 replicates to choose the best *K* value. Output files (Q-matrices) from replicated runs for the best *K* value were analyzed with the CLUMPP v1.1.2 (Jakobsson and Rosenberg 2007) software to identify common modes among replicates. By doing so, we selected *K* = 6 (Supplementary Fig. 1) and used the greedy algorithm with 100 random input orders to be tested and *G*′ pairwise matrix similarity statistics.

The FineStructure software enables capturing information provided by patterns of haplotype similarity. It summarizes this information in a coancestry matrix. We used the linked model, which harnesses LD information from the data (Lawson *et al.* 2012). The software first implements the ChromoPainter algorithm to reconstruct each haplotype using all individual haplotypes in the sample. The software then calculates the number of haplotype “chunks” used to reconstruct the recipient individual from each donor individual; the resulting matrix is called the linked coancestry matrix (Alexander *et al.* 2009; Lawson *et al.* 2012).

The variation in the resulting coancestry matrix was further explored via principal components analysis (PCA) approach implemented in the FineStructure software. In this model-based approach, the posterior probability of a population’s configuration is inferred using a Markov chain Monte Carlo (MCMC) implementation. The parameters convergence to the posterior distribution can be confirmed by comparing population memberships between 2 runs initialized with different random seeds. FineStructure then creates a Maximum a Posteriori set of populations as MCMC state and imposes a tree structure on them.

We also applied the concept of “super-individuals” built-in FineStructure. The approach allows some individual samples to be grouped. This approach allows the investigation of substructure details without additional computational costs. To refine the substructure among Brazilian samples, we grouped all other samples into one “super-individual” and proceeded to the FineStructure analysis anew.

### Diversity analysis

To assess diversity in Latino populations, we used haplotypes inferred in the phasing step to calculate the haplotype diversity parameter proposed by Nei and Tajima (1981) from windows of 10 markers using pegas R package (Paradis and Barrett 2010). We also estimated expected heterozygosity in PLINK.

### LD decay

We compared LD in the Latino populations to the populations of European, African, and Latin American ancestry. LD decay was estimated using PLINK as a function of LD by physical distance in kb. We filtered out only markers failing QC, thus keeping 196,749. We calculated the pairwise squared correlation coefficient (*r*^2^) for SNPs in a 100-kb window. SNP pairs were divided into 1 kb bins and *r^2^* was averaged within each bin.

### IBD segment detection and IBD score

Phased haplotypes were used to determine IBD segments sharing within populations through GERMLINE v1.5 (Gusev *et al.* 2009) software. We used 64 markers to extract matching seeds; a maximum allowed number of mismatching homozygous and heterozygous markers was set to 1; we also allowed extension from exact seeds using haplotypes rather than genotypes and allowed for the extension of the match beyond the slice end to the first mismatching marker. The IBD score was computed as the total length of IBD segments between 3 and 20 cM normalized by sample size $\left\{C_{2}^{2n}-n\right\}$, where *n* is the number of individuals in each group. Standard errors were calculated employing a weighted block-jackknife (Kunsch 1989; Busing *et al.* 1999) over 10 Mb segments, with 95% confidence intervals defined as IBD-score times ±1.96 the standard error.

### Estimation of ROH

ROH calling was performed for populations from Latin America and Native Americans using PLINK on LD pruned SNPs. To make array data (BR population) comparable to low-coverage WGS data (other populations in the dataset), we set the parameters following the guidelines in Ceballos, Hazelhurst, *et al.* (2018) and the findings in Howrigan *et al.* (2011). The number of heterozygotes allowed in an ROH call (–homozyg-window-het parameter) was set differently between technologies: we allowed no heterozygote site for array data since SNP array has low genotyping calling error rates (generally < 0.001) while allowing up to 3 heterozygotes in WGS data. We also examined only segments greater than 1 Mb in length. This is because WGS data systematically detects more short ROH (up to 1 Mb) than array data, and segments longer than 1 Mb correspond to true ROH originating from IBD (thus removing any LD effects) (Ceballos, Hazelhurst, *et al.* 2018). The analysis was conducted using the following list of parameters: –homozyg-snp 50, –homozyg-kb 1000, –homozyg-gap 1000, homozyg-widow-snp 50, –homozyg-window-missing 5, –homozyg-window-threshold, 0.05, and –homozyg-window-het 0/3 (microarray/WGS). Importantly, the above parameters are best suited for detecting autozygosity within the past 20 generations (Howrigan *et al.* 2011).

### Estimation of population effective size (*N*_e_)

We used the IBDNe software to estimate historical *N*_e_ (Browning and Browning 2015) in the Latin American population sample following the pipeline suggested by the authors for recently admixed populations, that is, we applied the haplotype-based Refined IBD method beforehand (Browning and Browning 2013) to account for population heterogeneity. Then, we performed the merging of gaps (*merge-ibd-gaps* script) to remove breaks and short gaps in IBD segments resulting from haplotype phase and genotype errors. We assumed a 30-year generation time.

### Integrated haplotype score

The integrated haplotype score was proposed by Voight et al. (2006) as a method to describe events of incomplete hard sweeps caused by recent positive selection. The idea is to harness the unusually long haplotype of low diversity caused by an allele that has undergone a fast increase in frequency. The iHS measures the amount of EHH at a given locus. We annotated alleles’ polarity (ancestral/derived status) beforehand. We computed iHS values for each Latino population separately and for 125 samples drawn from simulations (each one containing 80 diploid individuals) using the method implemented in selscan v1.3.0 (Szpiech and Hernandez 2014) from phased haplotypes. In this method, EHH is integrated with respect to genetic distance by linear interpolation between SNPs until EHH reached 0.05 in both directions from the core marker, otherwise, the SNP was skipped. Normalization is then performed to account for regional differences in allele frequencies. Normalized iHS has a mean of 0 and variance of 1, and selscan authors suggest that values that lie 2 variances away from the expected under neutral hypothesis signal positive selection (by this criterion, iHS < −2 would mean selection on the ancestral allele, while iHS > 2, represents selection on the derived allele) (Szpiech and Hernandez 2014). To correct for LD effects (including admixture LD), randomness from small sample sizes, and multiple testing, we compared scores from each Latino population to that of neutral simulations matched for the same number of tests.

### Demographic simulations

To generate empirical cut-off values for iHS, we performed iHS calculations on neutrally evolving individuals drawn from simulations. The demographic model used here is closely related to that of Mooney *et al.* (2018), except that in the present model all markers were neutral and the colonization bottleneck was more severe and lasted longer. The purpose is to generate long-range LD capable of yielding iHS signals comparable to the empirical genomic data.

Using the forward simulation implemented in the SLiM 3.6 software (Messer 2013), we performed 10 independent simulations of diploid individuals for a 10-Mb chromosome under a uniform recombination rate of 1 × 10^−8^ crossing-over events per chromosome per base per generation and mutation rate of 1.5 × 10^−8^ mutations per chromosome per base position per generation. Markers used in the downstream analysis are the neutral mutations generated that were still segregating as polymorphisms (did not reach fixation have a frequency greater than 0.05).

The simulation starts at 2.15 million years before the present to allow for a burn-in period to reach mutation-drift equilibrium. The simulation is then divided into 10 runs each of them beginning in the Out-Of-Africa (OOA) migration event. We assumed an effective population size of 10,000 individuals, and a reduction in size to 2,000 starting 50,000 years ago (reflecting the OOA event), followed by a recovery to 10,000 individuals 5,000 years ago (Gravel *et al.* 2011). The colonization bottleneck is assumed to occur 500 years ago by an admixture event with Europeans, which contribute 70% of the genomes to the admixture proportion, and the colonization entails a reduction in effective size to 100 individuals from 500 to 90 years ago and an inbreeding probability of 70% during this period (Mooney *et al.* 2018). The effective size then returned to 10,000. We assumed 30 years per generation. At the end of the simulations, 1,000 individuals were randomly sampled from each run, amounting to 10,000 individuals. The complete simulated dataset was further divided into 125 groups of 80 individuals containing approximately 12,000 markers each to perform iHS calculations.

### Time to the most recent common ancestor

As a proxy to the allele age, the time to the most recent common ancestor (TMRCA) was inferred assuming a star-shaped phylogeny, constant population size, and panmixia for putatively selected alleles as described in Voight *et al.* (2006) The probability that 2 chromosomes are homozygous (Pr[Homoz]) at a given genetic distance *r* from the selected site is:

$$\text{Pr}\left[\text{Homoz}\right]=e^{-2\mathrm{rgT}}$$

where *T* is the TMRCA in generations and *g* is the generation time in years. When 75% of the chromosome have recombined off of the haplotype under analysis (that is, Pr[Homoz] = 0.25), we assumed that the putatively selected haplotype reached its breakpoints. We then retrieve the recombination distance (*r*) from the GRCh37 genetic map and input it into the above formula for each locus. When the genetic distance was greater than 1.1 cM between recombination breaks (TMRCA ∼3,150 years before the present), we skipped TMRCA calculation, because an allele selected so recently would require a selection coefficient well above 5%, which is unrealistic (Kelley *et al.* 2006).

## Results

### Subjects and genotyping

After QC, 561 samples from Latin America (43 Native Americans, 85 Peruvians, 64 Mexicans, 94 Colombians, 104 Puerto Ricans, and 171 Brazilians), along with 1,165 worldwide samples were analyzed.

### Admixture analysis

After 10 ADMIXTURE runs with different seed values and by varying the number of ancestral clusters (*K*) from 2 up to 12, we selected *K* = 6 as the lowest CVE (CVE = 0.5776; Supplementary Fig. 1). All replicate runs had a pairwise similarity coefficient of 0.999.

**Fig. 1.**
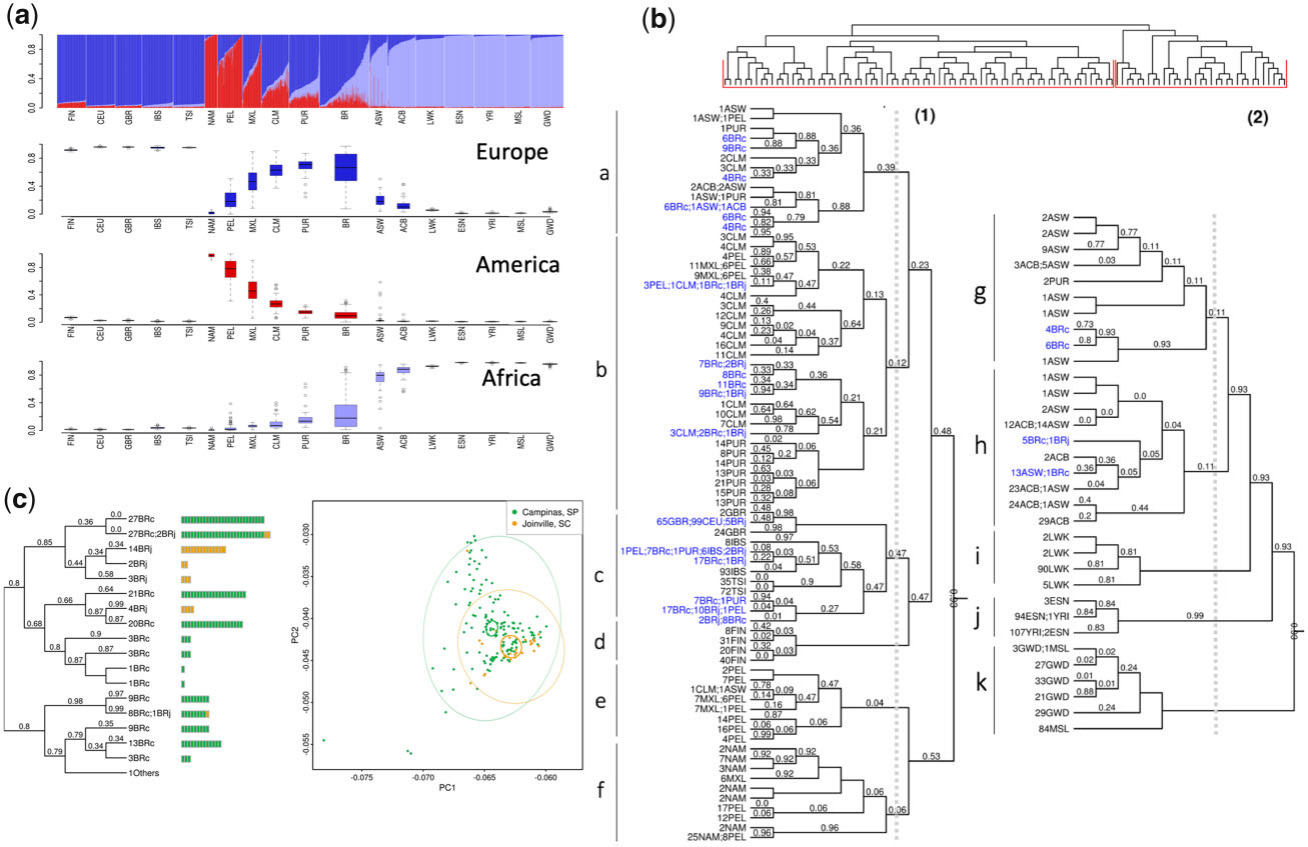
Admixture in Latino populations. a) ADMIXTURE analysis. Top panel: *K* = 3. Individuals are represented by vertical bars, the colors represent the estimated proportion of each cluster amounts to. Boxplots: genomic membership to each cluster: top: European ancestry; middle: Native American ancestry; and bottom: African ancestry. b) FineStructure tree of relationship across samples in the complete dataset. Posterior probability values below 1 are shown as branch labels. Dotted gray lines represent the cuts on the tree used to generate groups of distal clusters (a–k, see text). Edge labels show population membership and the number of individuals in each leaf. The dataset splits into 2 major clusters, according to non-African (1) and African (2) genomic predominance. Brazilians and Puerto Ricans are represented in both major clusters. Brazilians (in blue), however, are also more dispersed in distal clusters when compared to any other Latino Population, being the most dispersed admixed population, followed by ASW. BRc: samples from Campinas; BRj: samples from Joinville. c) Substructure in the Brazilian population. *Left panel*: FineStructure tree of relationship for Brazilian samples. Each colored bar corresponds to an individual, colors represent sample collection sites: green—Campinas (São Paulo State) and orange—Joinville (Santa Catarina State). Brazilian samples were subdivided into 18 leaves. Other populations were averaged and shown as super-individual (“1Others”). Right panel: PCA for PC1 and. PC2 on Brazilians. Inner ellipses are the 95% confidence ellipses for the barycenters of the groups. Outer ellipses are the 95% confidence ellipses for the groups.

The plots representing individuals within each population as a combination of optimal *K* ancestral components are depicted in Supplementary Fig. 2. We present a plot for *K* = 3 in Fig. 1a, wherein one can observe the 3 main continental contributors to the overall ancestry components: European, Amerindian, and African. The genetic structure of Latin American populations showed different mosaics from 2 or 3-way mixing from these 3 main clusters (top panel in Fig. 1a).

Supplementary Table 2 shows Latinos’ mean and standard deviations for broad European, African, and Native American ancestries. Southern/Southeastern Brazilians (BR) were the only Latin Americans to have greater African ancestry relative to Amerindian ancestry (proportions were as follows: 10.2% Amerindian, 24.9% African, and 64.9% European ancestry components). Brazilians also showed the greatest variability for European and African components, while Peruvians and Mexicans have a greater variance for the Native American component. Brazilians also had the highest diversity measured by haplotype diversity and expected heterozygosity (Table 2). Both measures agree in the ranking among samples: Puerto Ricans come after Brazilians in diversity and are followed by Colombians, Mexicans, and Peruvians, in descending order. The ancestry composition and diversity described here are consistent with other publications describing Brazilians (Pena *et al.* 2011; Giolo *et al.* 2012; Kehdy *et al.* 2015; Rodrigues de Moura *et al.* 2015) and other Latinos (Bryc *et al.* 2010; Mooney *et al.* 2018). Although useful in comparing populations and markers in the scope of this study, we advise that caution is necessary for interpreting this result, since filtering by markers from array panels generates ascertainment bias and inflates measures of diversity (Geibel *et al.* 2021).

**Table 2.** Diversity in Latino populations as measured by expected heterozygosity and haplotype diversity.

Population	Expected heterozygosity	Haplotype diversity
Brazilian	0.383972	0.8371
Puerto Rican	0.378918	0.8126
Colombian	0.377863	0.7991
Mexican	0.372061	0.7770
Peruvian	0.350500	0.7256

### FineStructure analysis

We performed a PCA (Supplementary Fig. 3) derived from the coancestry matrix obtained from FineStructure. Eigenvalues decrease rapidly with the increment of PC rank (Supplementary Fig. 4), with the first 5 eigenvectors explaining 84.8% of the variance present in the data. The tree generated from all samples includes 108 terminal groups (leaves) divided into 2 major clusters (highlighted in red in Fig. 1b) accounting for (1) prevalence of genomic components other than African and (2) prevalence of African composition (Fig. 1b).

Cluster 1 further splits into 6 groups (Fig. 1b—*a*–*f*): 2 generally admixed (Fig. 1b—*a* and *b*), 2 predominantly European (Fig. 1b—*c* and *d*), and 2 predominantly Native American (Fig. 1b—*e* and *f*). Brazilian samples occur in all the above-mentioned groups, except for the Native American-related clusters. Approximately 10% of Brazilian samples were assigned to cluster 2, the other 44% were in the European branches, and the remaining samples are grouped with generally admixed populations. The other 390 Latin American samples are essentially distributed in cluster 1 (except for 2 Puerto Rican samples). Conspicuously, Americans of African Ancestry in the United States (ASW), which is an admixed African-American population, also presented a pattern of high dispersion, although showing predominance in the African related cluster rather than in European clusters.

Since the Brazilian sample was collected in 2 different locations, we sought to inspect the population structure in more detail. The Brazilian set of samples was divided into 18 subgroups (Fig. 1c). The resulting tree and PCA plot proved to be sensitive enough to capture differentiation among the samples.

### LD decay

**Fig. 2.**
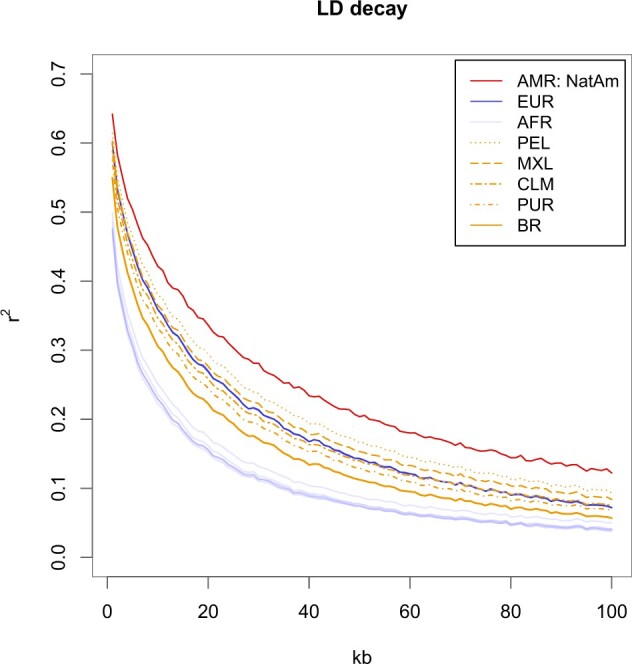
LD-decay plot. LD (*r^2^*) was estimated with PLINK software and plotted by population as a function of physical distance in kb. Latino populations are represented in yellow, the Native American population is represented in red, South European populations are represented in blue, and African populations are represented in purple. North-Europeans were not plotted to allow Latino populations to be better visualized.

LD, as measured by *r*^2^, showed the highest values in Native Americans, followed by Peruvians and Mexicans; while the lowest values were from the populations of African origin (Fig. 2). Other Latinos (CLM, PUR, and BR, in LD descending order) displayed less LD than South Europeans, but still more than African populations. The distribution of LD among Latinos follows closely the proportion of African and Native American ancestry.

### IBD segments sharing and IBD score

**Fig. 3.**
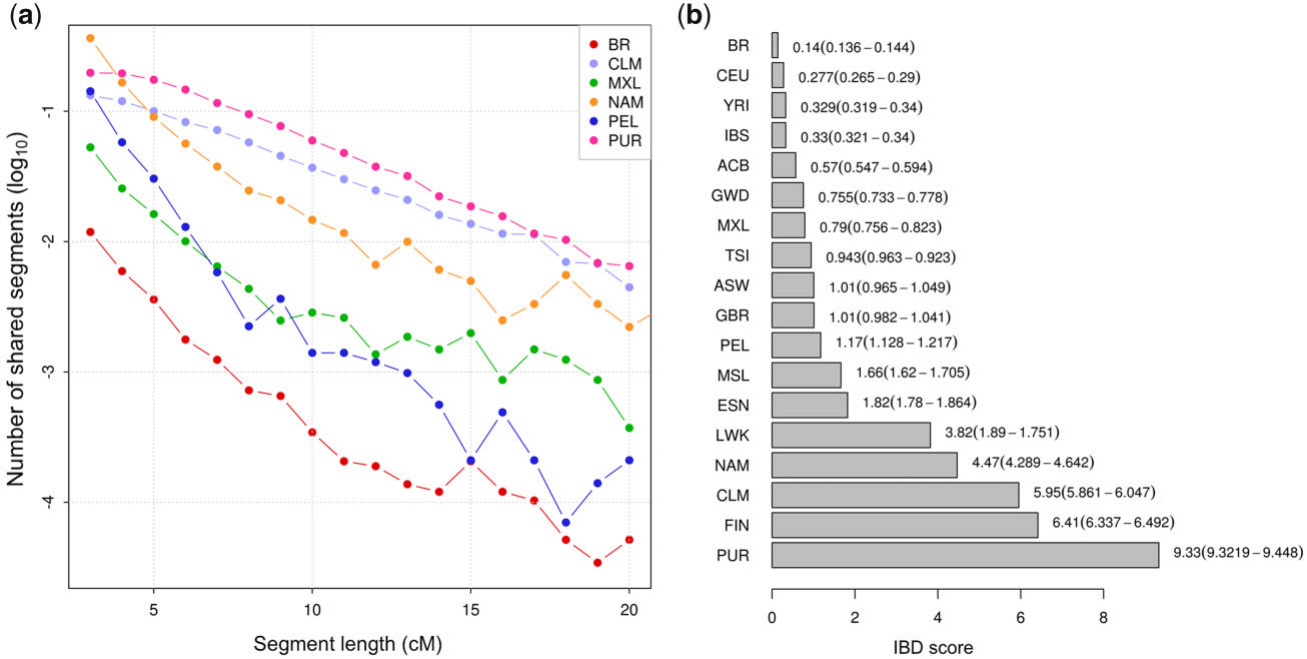
IBD sharing in Latinos and IBD scores for the whole dataset. a) Log_10_ of the number of pairwise IBD segments shared within each Latin American and Native American population by segment length, ranging from 2 to 20 cM. The values were normalized by the total number of pairwise comparisons. Segment lengths were approximated to the nearest integer number. b) IBD scores for all populations considered in the present study. Scores were calculated by computing the total length of all IBD segments between 3 and 20 cM and normalizing values by sample sizes. A total of 95% confidence intervals are depicted under parenthesis.

IBD analyses are summarized in Fig. 3. The number of IBD segments of different lengths shared by pairs of individuals in Latin American populations is shown in Fig. 3a. Puerto Ricans and Colombians had the highest IBD sharing values, above Native Americans, while Brazilians presented the lowest values. Peruvians and Mexicans had intermediate values. The IBD fragment length scores with the respective standard errors are shown in Fig. 3b. In the analyzed dataset, Brazilians had the lowest IBD score both in terms of fragment number and length, when compared to other worldwide populations. Conversely, Puerto Ricans exhibited the highest IBD scores in the whole dataset.

### Runs of homozygosity

We computed ROH for Latin and Native American populations, the total number of ROH in each population was: 5,551 in PEL, 4,575 in NAM, 2,555 in CLM, 2,342 in MXL, 2,190 in PUR, and 2,082 in BR. The median ROH length varied little among these populations, ranging from 1,293 (BR) to 1,335 kb (NAM), see Supplementary Fig. 5 for a representation of ROH sizes distribution (up to 15 Mb) across the whole dataset.

**Fig. 4.**
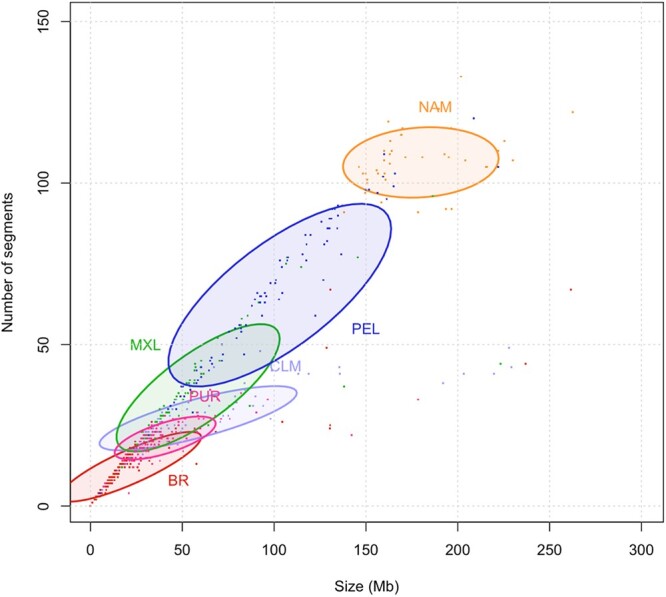
ROH in American populations. The number and the length sum of all ROH segments (Mb) per individual. Ellipses correspond to 50% confidence intervals for each population.

Nonetheless, Peruvians and Native Americans had an enrichment in long ROH, as depicted in Fig. 4. The plot shows the sum of segments by their total length in Mb for each population. Native Americans presented the highest count of large ROHs, while BR had the lowest.

### N_e_

**Fig. 5.**
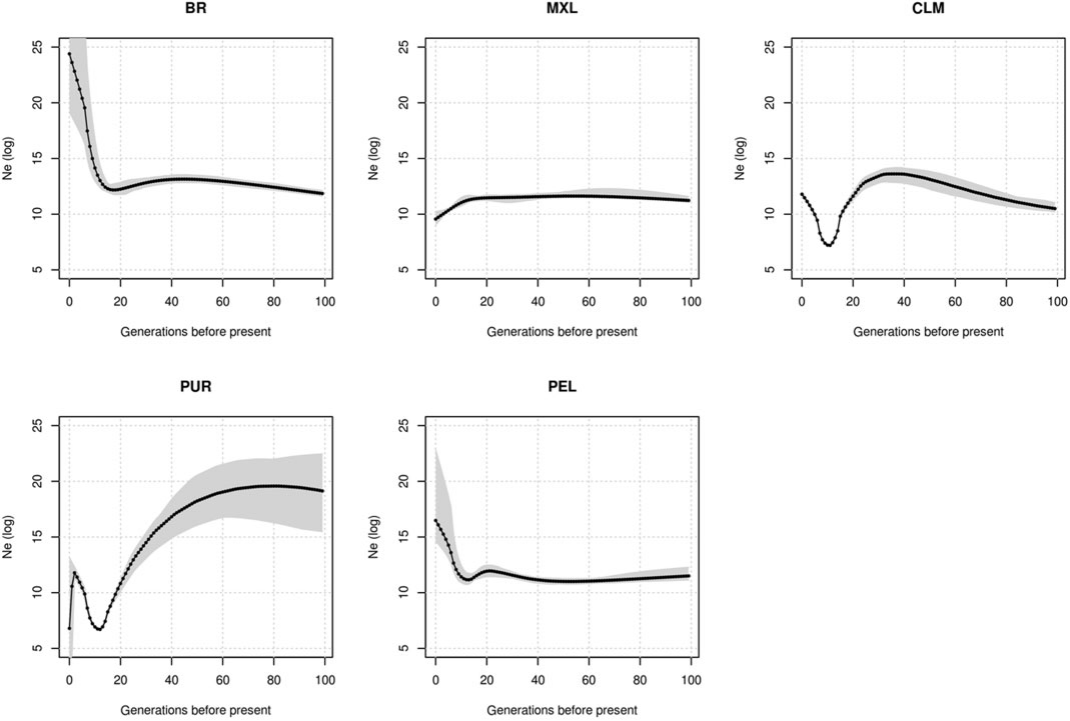
Effective population sizes (*N*_e_) in Latinos. IBDNe software was used to infer effective population size over the last 3,000 years. A total of 50% confidence intervals are represented as shaded regions. CLM and PUR show severe bottlenecks approximately 500 years ago and do not recover from these events. PUR effective size also shows a recent variation toward lower effective size, although estimates nearer the present tend to be inaccurate.

To further understand the IBD and LD patterns and their relation to the population history, we estimated the effective population size of the Latin American populations (Fig. 5). Our estimates are in agreement with those of Mooney and cols. for CLM, PUR, MXL, and PEL populations (Mooney *et al.* 2018). In effect, Colombians and Puerto Ricans have endured bottlenecks between 200 and 400 years ago, and neither have completely recovered from these events. Other Latino populations, on the other hand, had either a stable effective size (Mexicans) or underwent a size growth in the past 500 years (Brazilians and Peruvians).

### Demographic simulations and iHS

We pooled the iHS values results from the 125 groups generated by neutral simulations. Each group underwent iHS analysis with the same parameters used for the empirical data. After removing variants with MAF < 0.05 as advised by selscan authors (Szpiech and Hernandez 2014), we ended up with an average of 12,268 markers in each of these groups, therefore yielding a total of 1.53 million iHS calculations, which is approximately 1.63 times the total number of tests run in all empirical data from Latino populations (983,745). Although total scores surpassing the conventional threshold of selection (|iHS| > 2) in neutral simulations were only 1.12 times greater than the total found in all Latino populations (32,731 vs 29,175), these results indicate that LD and random sampling in small datasets seem to be important sources of false positives.

### iHS in empirical data

We used the iHS scores obtained from simulations (matched by the number of interrogated markers) to get empirical cut-offs for each Latino population. The iHS cut-off values and the number of remaining markers for each population were: −4.594/3.294 for Peruvians (134 markers remained); −4.749/3.382 for Mexicans (97 markers remained); −5.022/3.826 for Colombians (28 markers remained); −4.711/4.124 for Puerto Ricans (12 markers remained); and −4.783/4.214 for Brazilians (2 markers remained), for comparison between simulated and empirical data, see Supplementary Fig. 6.

**Fig. 6.**
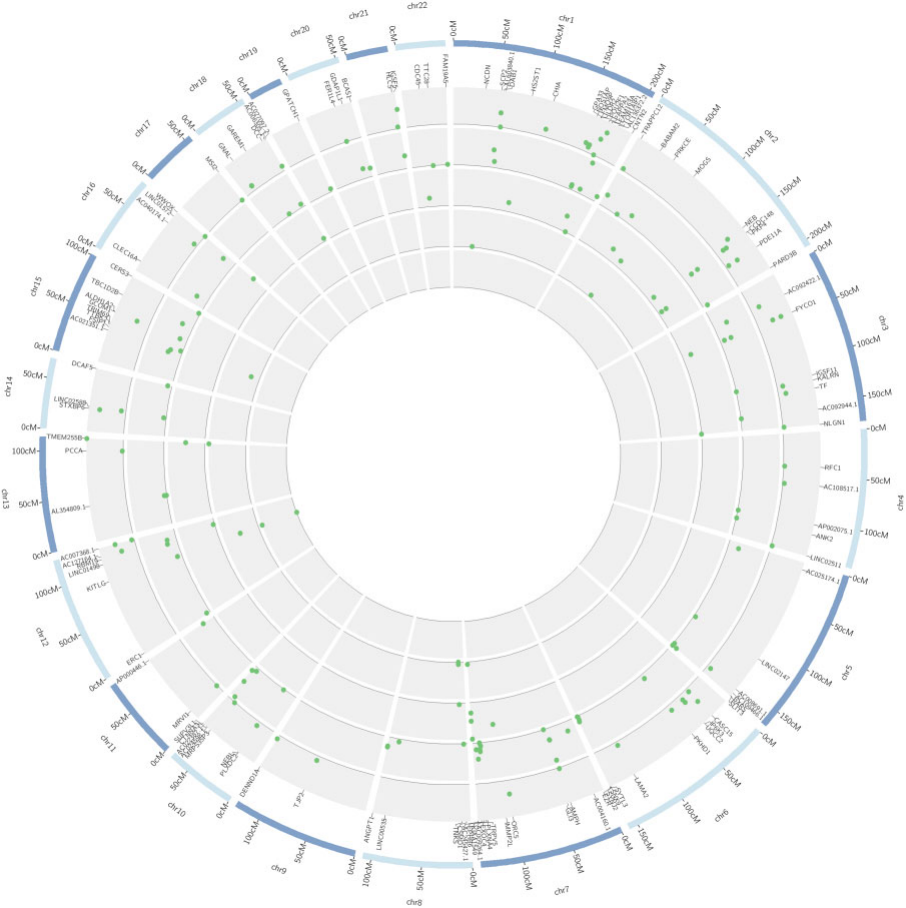
Circular plot of extended haplotype scores (iHS) in Latino populations. Internal to the human karyogram (blue) are the standardized iHS values represented by scatterplots. From the inner to outer circles: Brazilian, Puerto Rican, Colombian, Peruvian, and Mexican populations. Only genic iHS values surpassing the simulation thresholds for each population are displayed (see Supplementary Table 3 for detailed information on the markers).

Of the total of 244 markers resilient to comparison with simulations (26 of which were common to 2 or more populations), 142 were intronic (58%), 93 were intergenic (38%), and 9 were exonic (4%). From the total 119 genes (19 were common to 2 or more Latino populations) presenting signals that overcome simulation generated thresholds (Fig. 6 and Supplementary Table 3), a few were previously reported as targets of selection in other populations, such as *NCDN* (Voight *et al.* 2006), *ERC1* (Sabeti *et al.* 2007), *WWOX* (Sabeti *et al.* 2007; Choin *et al.* 2021), *TRIM69* (Sabeti *et al.* 2007; Tournebize *et al.* 2019), *PDE11A* (Sabeti *et al.* 2007; Choin *et al.* 2021), *KITLG* (Chen *et al.* 2015), *TNKS* (Eaaswarkhanth *et al.* 2020), *TRPV5* (Akey *et al.* 2004), *TF* (Akey *et al.* 2004), *EPHB6* (Akey *et al.* 2004), *IGSF11* (Chimusa *et al.* 2015), and *KALRN* (Chimusa *et al.* 2015). A gene from the aldehyde dehydrogenase (*ALDH1A2*) and immunoglobulin (*IGSF5*) families, a gene implicated in face shape variation (*GLI3*), as well as genes playing roles in the central nervous system (*ANK2*, *CNTN2*, *CSMD1*, *BCAS1*, *NLGN1*, and *TTBK2*) were also found as candidates of selection. Importantly, all the 273 signals (accounting for markers that were common to different populations) were resultant of a putative event of positive selection on the derived allele (positive values of iHS). We provide the list of intergenic markers putatively under selection, along with the nearest gene and allele TMRCA in Supplementary Table 4.

Noteworthy, genes that are hallmarks of adaptation in humans, such as *LCT*, *SLC24A5*, and *OCA2*, presented preliminary signals of selection in Latino populations but were not strong enough to overcome thresholds from simulated data.

### Time to the most recent common ancestor

We also sought to estimate the allele ages of the relevant selection signals by using the approach of Voight *et al.* (2006) (Supplementary Tables 3 and 4). Alleles in Puerto Ricans and Peruvians had an overall lower mean age (17,608 and 18,310 years, respectively), while Mexicans and Colombians had consistently higher mean allele ages (44,322 and 77,102 years, respectively). BR population had only 1 region with signals exceeding simulations, and the TMRCA for this event was 15,484 years before the present.

## Discussion

In the past few years, there has been much interest in admixed Latin American populations (Mao *et al.* 2007; Bryc *et al.* 2010, 2015; Montinaro *et al.* 2015; Santos *et al.* 2016; Mooney *et al.* 2018), but there are still few studies on how Latino populations compare among them in genetic terms. Mooney *et al.* analyzed Hispanic populations (from Puerto Rico, Mexico, Colombia, Peru, and Costa Rica) comprehensively regarding demographic histories and the impact of isolation and consanguinity on haplotype features, such as LD (also evaluated by Bryc *et al.* 2010), IBD sharing and ROH. The present study is, to date, the first report on these measures to include Brazilians in comparison to other Latino populations.

Here we found that the ancestral composition of contemporary Latinos agrees with previous reports (Fig. 1 and Supplementary Table 1) (Bryc *et al.* 2010; Santos *et al.* 2016; Martin *et al.* 2017). Latinos showed substantial variability in European and, to a lesser extent, Native American ancestry (except within Puerto Ricans) among and within each population, while African ancestry presented moderate to negligible variability in all populations, except for Brazilians (Supplementary Table 2). Of note, the mean and variance of markers outside the autosomes may diverge from the genomic landscape depicted here (Risch *et al.* 2009; Bryc *et al.* 2010; McHugh *et al.* 2016; Kehdy *et al.* 2015), and genealogies of uniparental markers were shown to display genetic heterogeneity (Alves-Silva *et al.* 2000; Carvalho-Silva *et al.* 2001; Pena *et al.* 2009; Bernardo *et al.* 2014).

The findings presented here are consistent with the Brazilian demographic records. An estimate of 2.5 million indigenous people, from various ethnicities, lived in Brazil (IBGE 2012; Salzano and Sans 2014) when the Portuguese settlers first arrived. Portuguese migrants reached around 500 thousand in the early 19th century period. Also, from 1650 to 1850, Brazil was the destination of 4 million of the 9–11 million Africans forced to migrate to the Americas (Alencastro 2000), with important influxes in the 1780–1850 period (1.7 million people) (Alencastro 2000), being the single greatest destination of the trans-Atlantic slave trade. Brazilians had their Native American component eroded by the displacement and massacre of indigenous people, and by the following influx of African and European people.

Hispanic America, in contrast, had a lower influx of African people (1.6 million throughout several former colonies) and most arrivals occurred in the 16th and 17th centuries (Alencastro 2000), although agricultural production in Caribbean possessions largely depended on African forced labor (Puerto Ricans had an African ancestry mean close to that of Brazilians). Accordingly, Gouveia *et al.* (2020) found the time of admixture events to be older in Puerto Ricans, Colombians, and African Peruvians, compared to Brazilians. In effect, Hispanics analyzed here displayed greater Native American and lower African ancestries than Brazilians.

Another difference in Spanish America is that large centers were densely populated by the time the Spanish arrived, e.g. Tenochtitlan was larger than most, if not all, European cities and was among the largest cities in the globe at the time, possibly reaching 300,000 inhabitants (Elliott 2008). Similarly, the Inca capital city, Cuzco, had an estimated 100,000 inhabitants by 1532 (Bethell 2008). Accordingly, here we found Peruvian and Mexican populations to be more indigenous-related.

We observed that the amount of the Native American contribution readily translated to the LD patterns in the current Latino populations (Fig. 2), a finding previously described by Bryc *et al.* (2010). LD decay in the Brazilian population departs from the cluster formed by other Latino populations, which is consistent with its high proportion of African and low proportion of Native American components. However, Puerto Ricans share similar proportions of both Amerindian and African ancestries with Brazilians, so we propose that other factors might impact LD, though marginally. Puerto Ricans had the highest IBD sharing and score, and have lived on an island and thus did not experience population growth comparable to Brazilians. Puerto Ricans were also found to have smaller founder sizes than other Latinos (Browning *et al.* 2018). Conversely, the large Brazilian effective size and fast population growth, relative to the other Latinos, act by retaining haplotype diversity and thus further relaxing LD.

These differences between Puerto Ricans and Brazilians, nonetheless, account for a minimal fraction of the LD pattern, if any. We found a smaller average haplotype size and higher genetic diversity in Brazilians. FineStructure and ADMIXTURE analyses are in agreement with these findings and corroborate previously published studies (Gusev *et al.* 2009; O’Connell *et al.* 2014). Although the FineStructure clustering approach showed Brazilians and Puerto Ricans to be more dispersed throughout the computed tree, large clusters combining Brazilian and Colombian, or Brazilian and Peruvian populations suggest a large number of shared haplotypes within these groups.

Because Brazilians were highly dispersed in comparison to Hispanics, we proceeded to evaluate if regional differences could partially be accountable for this phenomenon. Indeed, we were able to pinpoint a moderate structure among the geographically distinct groups inside Brazil (Fig. 1c). Although the general distribution of the genotypes mostly overlaps on the PCA plot, the populations’ means differ slightly. Several estimates have been made regarding the degree of regional genetic discrepancy in Brazil, and the emerging trend is that urban areas are rather similar and rural and isolated settlements are less so (Shifman *et al.* 2003; Wall and Pritchard 2003; Gusev *et al.* 2009; O’Connell *et al.* 2014). Although Brazilian samples were collected in 2 urban centers only 366 miles apart, we were still able to capture the divergence between them. FineStructure analysis applied for Brazilians ascertained samples into 2 major clusters, which correspond to pronounced African and European/American ancestry roots. We assert that this difference is demographical rather than purely geographical, 87% of the population of Joinville is auto-declared to be White, while in Campinas this proportion was 78.6% by 2010 (IBGE 2010b).

Interestingly, IBD measures captured nuances in demographic histories that LD decay did not. Although Native Americans were the population with the highest LD, Puerto Ricans tended to have the greatest IBD sharing, followed by Colombians. Puerto Ricans also had the highest IBD score; this extensive enrichment of IBD has been described in an earlier study, which also found similar results for Colombians (Mooney *et al.* 2018). Geographically isolated populations (from mountainous regions or circumscribing areas as islands) and/or those that have endured bottlenecks and isolation are more prone to share more and larger IBD segments. Severe bottlenecks are prevalent events in the foundation of different Latin American populations and should be taken into account when investigating genetic variation and disease.

Mexicans, Peruvians, and Brazilians did not endure severe bottlenecks (Fig. 5) at the beginning of their mixing. Furthermore, Brazil and Peru experienced population effective size growth afterward. Brazilians had the least net number and total length of the shared IBD segments among worldwide populations. The IBD values observed in Brazilian samples suggest high haplotype diversity and short and a relatively old founder event, followed by quick population growth, and a large actual effective population size (Pemberton *et al.* 2012).

Although undergoing the most severe bottlenecks, Colombians and Puerto Ricans do not show enrichment in large ROH (Fig. 4 and Supplementary Fig. 5), as admixture increased genetic diversity, a phenomenon described in population isolates from Colombia and Costa Rica (Mooney *et al.* 2018). Native Americans, on the other hand, also experienced bottleneck but not admixture, and have genomes enriched for large ROH segments, and Peruvians seem to follow this pattern for their prevailing Native American ancestry.

Extended haplotypes probably originating from events of the recent selective sweep were abundant in Latino populations (29 thousand markers exhibit signals inferred by iHS in this dataset). However, by comparing iHS values obtained from neutrally evolving simulation data for the same number of tests in each population, the remaining signals drop to 0.84% (244) of the original candidates. This confirms that long-range LD and random sampling can yield extended homozygosity and bias positive selection inspection. Our approach could partially counter these effects, since there is still less confidence in iHS estimates from small-sized samples, such as Mexicans (64), Peruvians (85), and Colombians (94). Nonetheless, the finding of Peruvians baring the most robust signals agrees with the hypothesis that signals are originating from recent adaptation, given the greater amount of Native American ancestry in this population.

While reducing the number of iHS signals to a more reasonable and reliable set of markers, the simulation approach used to produce cut-offs may be too rigorous, since Brazilians had a single region overcoming the thresholds, as well as signals in genes well-described as targets of selection were discarded after comparing to simulated individuals.

While there is a fair number of genes found here that were also found as selection targets in other studies, there are still many genes not previously linked to selection, possibly representing adaptation during the colonization of the American continent by humans beginning at least 15 thousand years ago (Reich *et al.* 2012). Interestingly, many TMRCA estimates for alleles putatively under selection are consistent with the timeframe of human settlement of America (Supplementary Tables 2 and 3). Peruvian and Puerto-Ricans populations’ mean estimates of TMRCA are also in line with this hypothesis. One must note, however, that the implementation of the TMRCA calculation presented here requires many simplifying assumptions, such as a star-shaped phylogeny, constant population size, and panmixia. Using simulations, Kelley *et al.* estimate that the TMRCA values as calculated by this method are about half the true TMRCA (Kelley 2012). Assuming this is true for our analyses, only a few signals would match the period of the first American settlements.

In conclusion, Latin American populations diverge in ancestral composition, diversity, and key haplotype features. We advise testing LD or using the above-mentioned estimates of LD when performing association analyses. Brazilians were the most heterogeneous admixed population and are second only to African populations concerning LD decay. Importantly, we were able to detect differences in urban populations from Brazil, suggesting less homogeneity than previously suggested. These observations are important for future genetic studies since the heterogeneity of this population makes it especially challenging to conduct association studies. We must take care when extrapolating findings of association studies to admixed populations. At the same time, the use of deeper sequencing methods aimed at finding rare variants may be a promising strategy to fill the gaps of missing heritability. Finally, we must be sure to explore the heterogeneity and benefits of the admixture mapping approach, whenever it is appropriate.

## Data availability

The complete dataset, including Brazilian samples, can be found at https://dx.doi.org/10.6084/m9.figshare.19640322 (last accessed May 9 2022). The above dataset represents the combined individuals in binary pedigree (bed/bim/fam) format, as well as VCF, and transposed pedigree (TPED) formats. Code used in analyses is available at https://github.com/soulsacross/Cruz-and-Ananina-2022 (last accessed May 9 2022).

Supplemental material is available at *G3* online.

## Supplementary Material

jkac111_Supplemental_Material

jkac111_Supplemental_Figure_S1

jkac111_Supplemental_Figure_S2

jkac111_Supplemental_Figure_S3

jkac111_Supplemental_Figure_S4

jkac111_Supplemental_Figure_S5

jkac111_Supplemental_Figure_S6

jkac111_Supplemental_Table_S1

jkac111_Supplemental_Table_S2

jkac111_Supplemental_Table_S3

jkac111_Supplemental_Table_S4
