# Supplementary material for: Demographic history differences between Hispanics and Brazilians imprint haplotype features
Source: G3 (Bethesda). 2022 May 2;12(7):jkac111. doi: 10.1093/g3journal/jkac111 (PMC9258545; doi:10.1093/g3journal/jkac111)
Supplement: jkac111_Supplemental_Material [file jkac111_supplemental_material.docx]

**Legends to Supplementary material**

**Supplementary Fig. S1**. **Cross-validation (CV) error.** CV errors calculated by ADMIXTURE for K varying from 1 to 12.

**Supplementary Fig. S2.** Mean ancestral components inferred by ADMIXTURE analysis. This analysis was performed using 176,390 SNPs across the autosomes. K = 6 had the lowest cross-validation error and thus was selected to represent ancestral components. The six ancestry components inferred for the dataset are distributed as follows: two European components comprising a “Northern,” which included primarily Finnish samples; and a “Southern,” which included Italian Tuscan and Spanish Iberian populations; three African components comprising three origins, here embodied in the Luhya (Kenya), Yoruba/Esan (Nigeria), and Mandinka (Gambian in Western Division, the Gambia) peoples; and an Amerindian component. **A)** Each bar represents an individual along the x-axis, while the y-axis depicts the mean proportional ancestry estimate in K proportion. **B)** Population averages for each K value. Population labels can be found in the main text's Table 1.

**Supplementary Fig. S3. PCA analysis from FineStructure results.** Plot of the PC1 to PC2 principal components. PC1 and PC2 together explain about 72.2% of the total variation. Population labels are in Table 1.

**Supplementary Fig. S4. Principal component (PC) rank.** Eigenvalues associated with the 15 first PCs (axes of variation) derive from the PCA analysis.

**Supplementary Fig S5. Distribution of ROH lengths across the whole dataset.** The plot is truncated in 15Mb.

**Supplementary Fig S6. Simulation and empirical iHS values comparisons. A)** Comparison of LD decay in Peruvians (PEL) and Brazilians (BR), populations with the greater and least LD in Latinos, respectively; compared with simulation (SIMULATION). Simulations with high long-range LD were generated to yield iHS values comparable to genomic empirical data. **B)** to **D)** Comparison between iHS values from simulations (orange) and Latino populations (black) for Puerto Ricans, Mexicans, Brazilians, Colombians, and Peruvians, respectively.

**Supplementary Table S1.** The average distance (in bp) along each chromosome for analyzed markers, the standard deviation for chromosomes is 2,226bp.

**Supplementary Table S2**. Average ancestries as estimated by ADMIXTURE with K=3. Latino populations means and standard deviation for major ancestral components.

**Supplementary Table S3.** Genic markers identified in putatively positive selection using iHS. TSS: transcription starting site, TMRCA: time to the most recent common ancestor. YBP: years before the present.

**Supplementary Table S4.** Intergenic markers identified in putatively positive selection using iHS. TSS: transcription starting site, TMRCA: time to the most recent common ancestor. YBP: years before the present**.**
