# Supplementary figures and images for: Demographic history differences between Hispanics and Brazilians imprint haplotype features
Source: G3 (Bethesda). 2022 May 2;12(7):jkac111. doi: 10.1093/g3journal/jkac111 (PMC9258545; doi:10.1093/g3journal/jkac111)

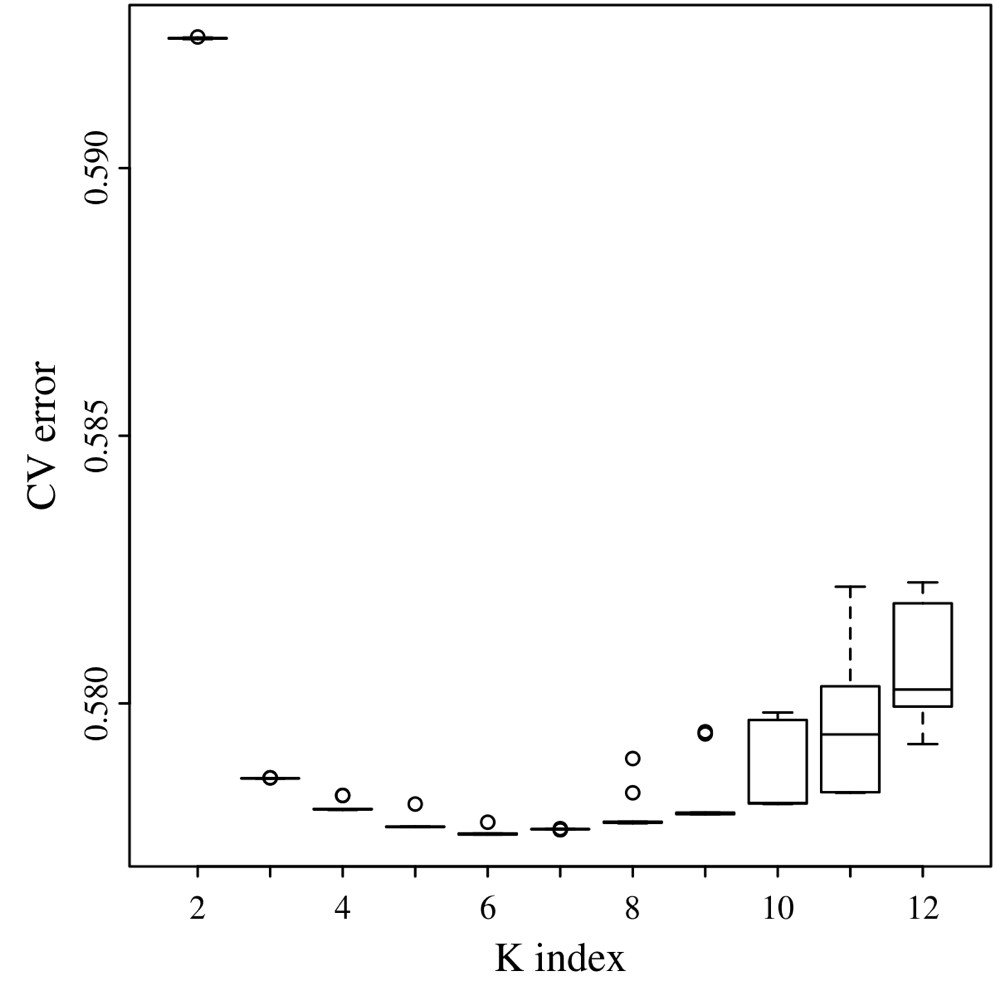

Supplement: jkac111_Supplemental_Figure_S1 [file jkac111_supplemental_figure_s1.jpeg]

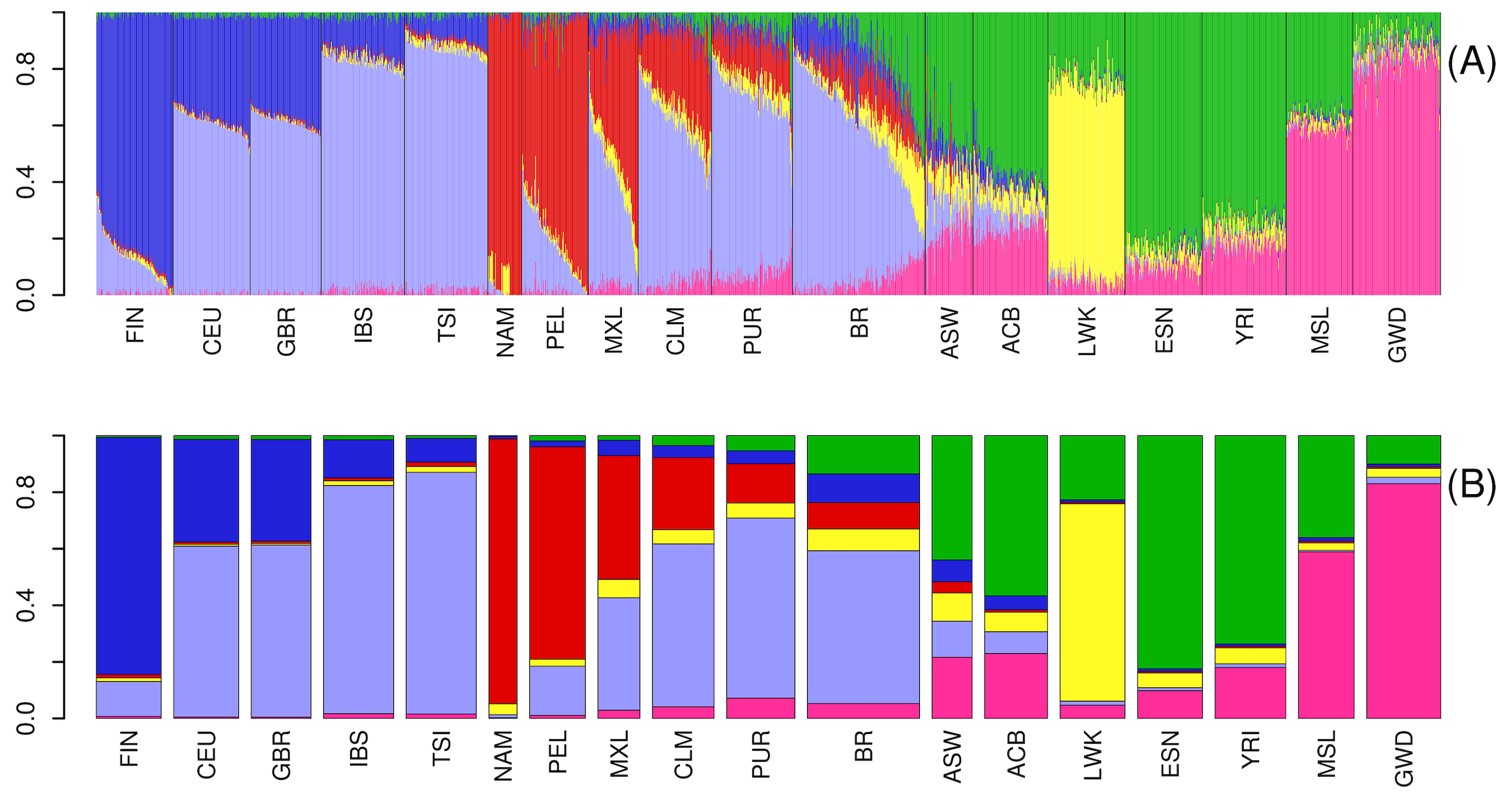

Supplement: jkac111_Supplemental_Figure_S2 [file jkac111_supplemental_figure_s2.jpeg]

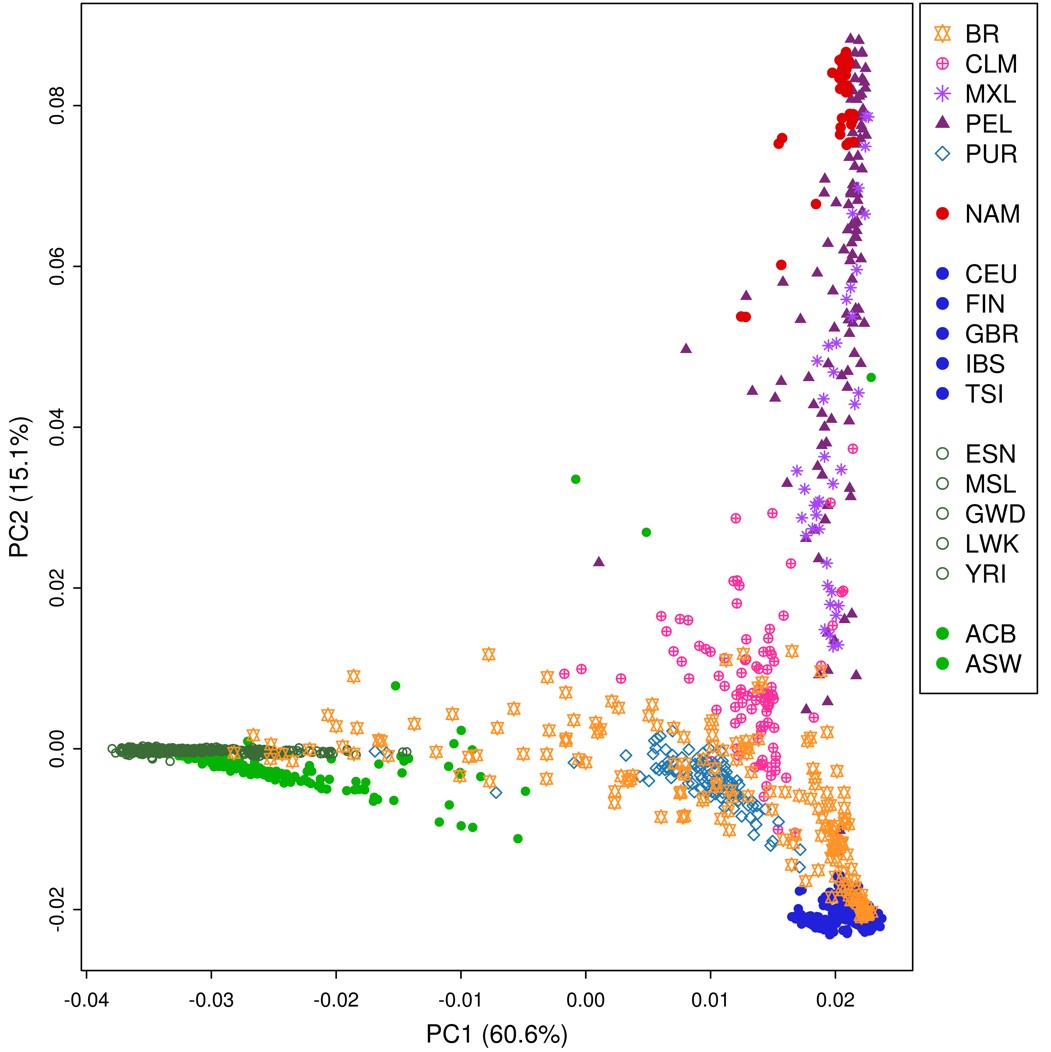

Supplement: jkac111_Supplemental_Figure_S3 [file jkac111_supplemental_figure_s3.jpeg]

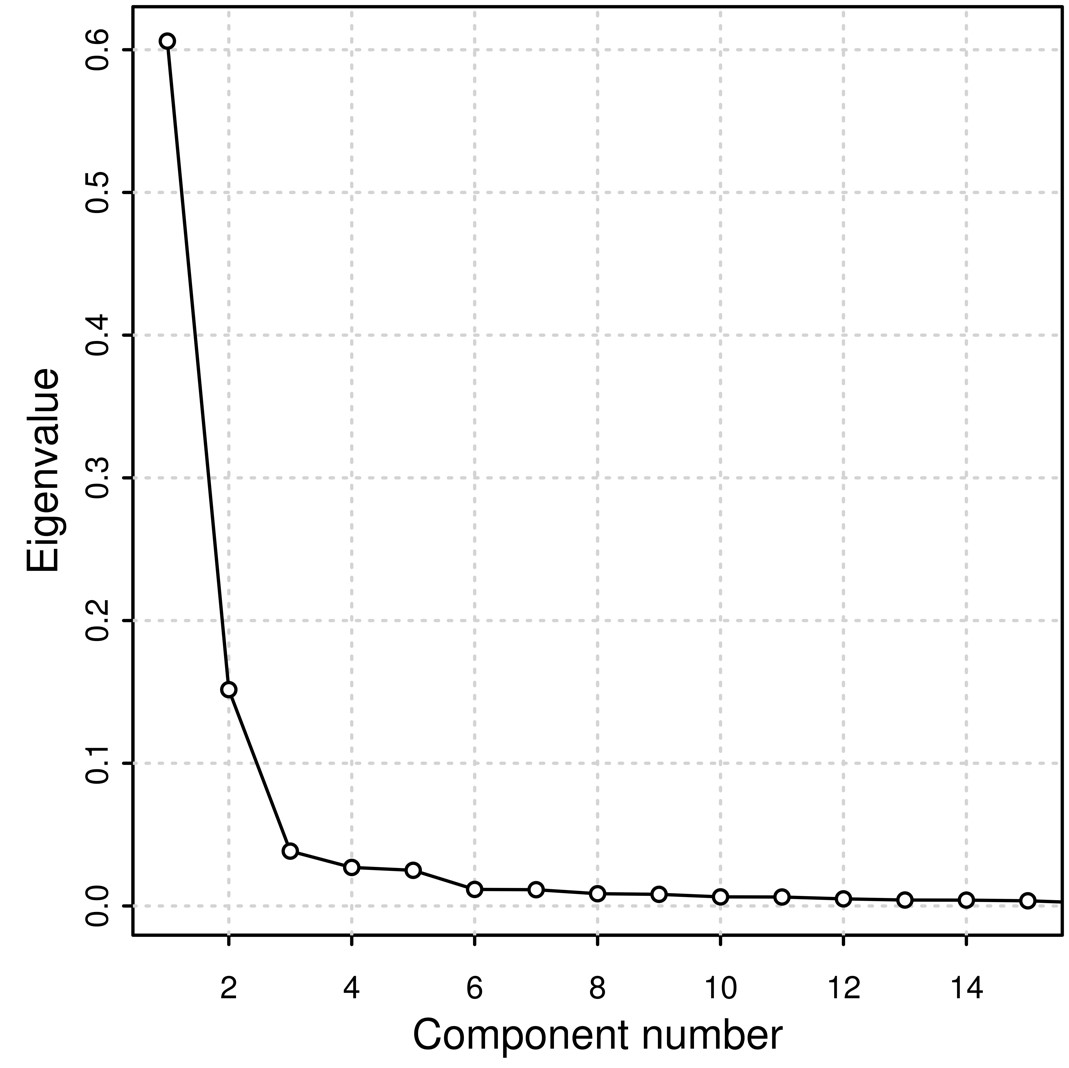

Supplement: jkac111_Supplemental_Figure_S4 [file jkac111_supplemental_figure_s4.jpeg]

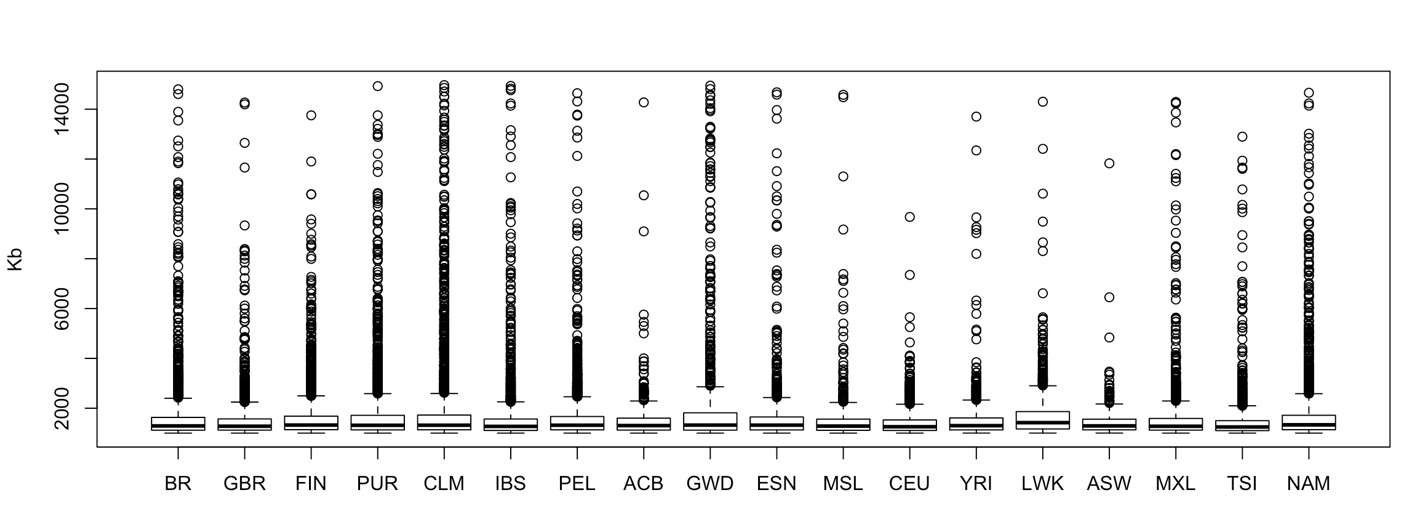

Supplement: jkac111_Supplemental_Figure_S5 [file jkac111_supplemental_figure_s5.jpeg]

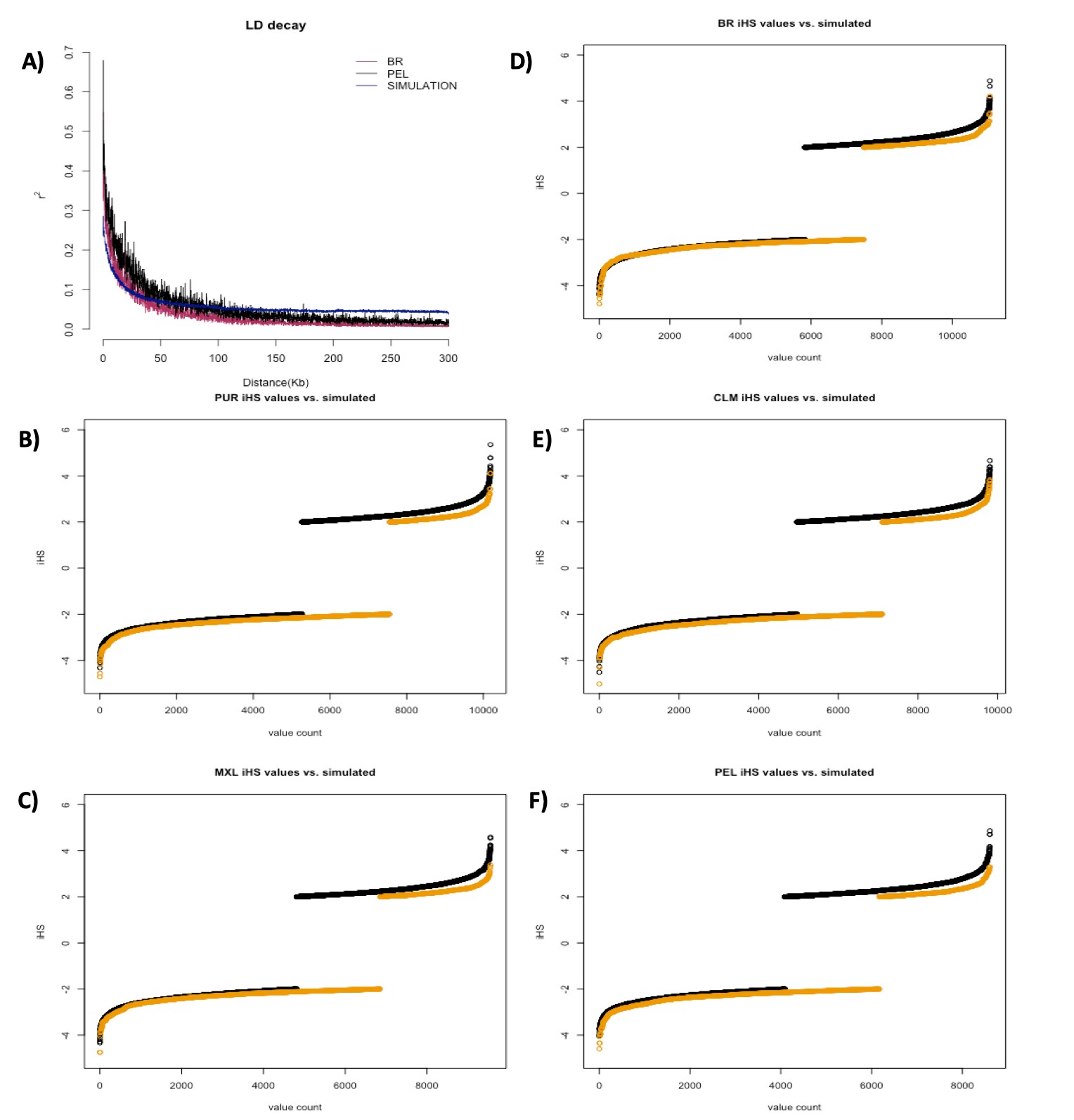

Supplement: jkac111_Supplemental_Figure_S6 [file jkac111_supplemental_figure_s6.jpeg]
