# Supplementary material for: Demographic history differences between Hispanics and Brazilians imprint haplotype features
Source: G3 (Bethesda). 2022 May 2;12(7):jkac111. doi: 10.1093/g3journal/jkac111 (PMC9258545; doi:10.1093/g3journal/jkac111)
Supplement: jkac111_Supplemental_Table_S1 [file jkac111_supplemental_table_s1.pdf]

Supplementary Table S1. Average distance (in bp) along each chromosomem. Standard deviation among chromosomes is 2,226bp.

Chromosome	Average distance (bp)
1	15712
2	15423
3	14872
4	14717
5	14863
6	14499
7	15249
8	13781
9	15774
10	11391
11	13353
12	13914
13	12189
14	13565
15	14439
16	15248
17	17126
18	12730
19	22121
20	12151
21	11875
22	14159
