# Supplementary material for: Demographic history differences between Hispanics and Brazilians imprint haplotype features
Source: G3 (Bethesda). 2022 May 2;12(7):jkac111. doi: 10.1093/g3journal/jkac111 (PMC9258545; doi:10.1093/g3journal/jkac111)
Supplement: jkac111_Supplemental_Table_S2 [file jkac111_supplemental_table_s2.pdf]

Supplementary Table S2. Average ancestries as estimated by ADMIXTURE with K=3. Latino populations means and standard deviation for major ancestral components.

	Average ancestries (K=3)			Standard Deviation		
**Population**	**Native American**	**European**	**African**	**Native American**	**European**	**African**
Peruvian	0.773	0.193	0.034	0.151	0.096	0.031
Mexican	0.499	0.447	0.054	0.165	0.140	0.018
Colombian	0.285	0.622	0.093	0.085	0.109	0.042
Purto Rican	0.161	0.689	0.151	0.034	0.086	0.050
Brazilian	0.102	0.649	0.249	0.049	0.277	0.105
