# Supplementary material for: Demographic history differences between Hispanics and Brazilians imprint haplotype features
Source: G3 (Bethesda). 2022 May 2;12(7):jkac111. doi: 10.1093/g3journal/jkac111 (PMC9258545; doi:10.1093/g3journal/jkac111)
Supplement: jkac111_Supplemental_Table_S3 [file jkac111_supplemental_table_s3.pdf]

Supplementary Table S3. Genic markers identified in putatuvely positive selection using iHS. TSS: transcription starting site, TMRCA: time to most recent common ancestor. YBP: years before present.

Chromosome	Position (bp)	rsID	Gene	Exon/Intron #	Area	TSS Distance (bp)	Normalized iHS	Population	TMRCA (YBP)
chr1	36027711	rs2074676	NCDN	4	INTRON	4318	4.18059	PUR	7172
chr1	53480745	rs7529265	SCP2	11	INTRON	87787	4.04565	PEL	10053
chr1	55984933	rs12757934	AL603840.1	1	INTRON	189972	3.85406	MXL	26226
chr1	55984933	rs12757934	AL603840.1	1	INTRON	189972	3.56552	PEL	17786
chr1	57535198	rs3738556	DAB1	5	INTRON	221539	3.39702	MXL	86232
chr1	87447902	rs10782588	HS2ST1	1	INTRON	67545	4.03481	CLM	12843
chr1	111857485	rs2820072	CHIA	4	INTRON	4489	3.63988	MXL	24077
chr1	162103270	rs10494368	NOS1AP	1	INTRON	63704	3.66045	MXL	10499
chr1	162106150	rs10918732	NOS1AP	1	INTRON	66584	3.66045	MXL	10499
chr1	166594960	rs486929	FMO9P	5	INTRON	13253	3.55818	MXL	16588
chr1	166876079	rs7552562	ILDR2	2	INTRON	1238	3.44066	PEL	10223
chr1	167126250	rs869714	GPA33	1	INTRON	9466	3.64324	MXL	32407
chr1	167198536	rs1021621	POU2F1	1	INTRON	8409	3.51325	PEL	7358
chr1	176453688	rs2223579	PAPPA2	1	INTRON	21381	3.39486	MXL	6341
chr1	176459709	rs742473	PAPPA2	1	INTRON	27402	3.96167	MXL	NA
chr1	179751937	rs7531276	FAM163A	1	INTRON	39639	3.48654	PEL	4603
chr1	179892125	rs1298479	TOR1AIP1	10	EXON	40678	4.17398	CLM	20962
chr1	179892125	rs1298479	TOR1AIP1	10	EXON	40678	4.23861	MXL	8967
chr1	179892125	rs1298479	TOR1AIP1	10	EXON	40678	4.70551	PEL	4726
chr1	188076020	rs4651434	AL136372.2	3	INTRON	93247	3.83903	CLM	255134
chr1	205027737	rs2275697	CNTN2	5	EXON	15485	3.53278	PEL	14785
chr2	3404845	rs12476309	TRAPPC12	2	INTRON	21388	3.45232	MXL	35301
chr2	3404845	rs12476309	TRAPPC12	2	INTRON	21388	3.8123	PEL	10453
chr2	28205581	rs7577342	BABAM2	4	INTRON	91922	3.33967	PEL	15831
chr2	46232372	rs2595222	PRKCE	8	INTRON	353459	3.65082	PEL	15157
chr2	54056574	rs805439	GPR75-ASB3	1	INTRON	30723	4.09045	CLM	NA
chr2	74690378	rs1063588	MOGS	3	EXON	2159	4.23914	PUR	11443
chr2	74690378	rs1063588	MOGS	3	EXON	2159	3.98976	CLM	9977
chr2	152574284	rs4413159	NEB	9	INTRON	16717	3.97729	MXL	17941
chr2	152574284	rs4413159	NEB	9	INTRON	16717	3.96237	CLM	17417
chr2	159249741	rs10190089	CCDC148	1	INTRON	63524	4.00002	PEL	3684
chr2	159423872	rs10933492	PKP4	2	INTRON	110261	3.6859	PEL	4099
chr2	159439965	rs6437190	PKP4	3	INTRON	126354	3.68084	PEL	4099
chr2	159447318	rs2108217	PKP4	3	INTRON	133707	3.68084	PEL	4099
chr2	159460150	rs1476672	PKP4	4	INTRON	146539	3.68073	PEL	4099
chr2	159466149	rs3771643	PKP4	4	INTRON	152538	3.63611	MXL	11843
chr2	159525930	rs2158831	PKP4	16	INTRON	212319	3.77177	MXL	16184
chr2	178492722	rs6730406	PDE11A	20	EXON	444760	3.91272	CLM	11404
chr2	178493770	rs6734426	PDE11A	20	EXON	443712	3.83258	MXL	11404
chr2	178498015	rs10469714	PDE11A	19	INTRON	439467	4.03356	CLM	11404
chr2	178498015	rs10469714	PDE11A	19	INTRON	439467	3.49699	MXL	11404
chr2	206144833	rs7606468	PARD3B	16	INTRON	734110	3.51346	PEL	3251
chr3	24929802	rs10865801	AC092422.1	4	INTRON	200392	3.89337	PEL	16051
chr3	25173349	rs6804502	AC092422.1	5	INTRON	443939	3.67658	MXL	13822
chr3	45993606	rs4682799	FYCO1	14	INTRON	43701	3.91366	MXL	17010
chr3	45993606	rs4682799	FYCO1	14	INTRON	43701	3.49043	PEL	8763
chr3	45993973	rs1552489	FYCO1	14	INTRON	43334	4.02788	CLM	97503
chr3	45993973	rs1552489	FYCO1	14	INTRON	43334	4.19675	MXL	17010
chr3	45993973	rs1552489	FYCO1	14	INTRON	43334	3.78582	PEL	8763
chr3	118656515	rs7633584	IGSF11	1	INTRON	167553	3.30479	PEL	NA
chr3	124385942	rs2289420	KALRN	14	EXON	82436	3.57441	MXL	227202
chr3	133499063	rs4854762	TF	17	EXON	34263	3.63379	MXL	109870
chr3	157077929	rs2173824	AC092944.1	2	INTRON	184871	3.31738	PEL	18968
chr3	173817935	rs6799153	NLGN1	3	INTRON	515590	3.82999	CLM	20485
chr3	173841089	rs9862758	NLGN1	3	INTRON	538744	3.43014	MXL	17582
chr4	39301334	rs3733284	RFC1	21	INTRON	66637	3.41794	MXL	12645
chr4	60041409	rs2334163	AC108517.1	1	INTRON	22857	3.43724	MXL	58662
chr4	102945076	rs2631265	AP002075.1	2	INTRON	12984	3.3257	PEL	89705
chr4	114058447	rs7664966	ANK2	1	INTRON	87562	3.32157	PEL	6923
chr4	137878262	rs17049175	LINC02511	2	INTRON	255691	3.41916	MXL	82924
chr4	137886834	rs7695609	LINC02511	2	INTRON	247119	3.41916	MXL	82924
chr5	7924914	rs179171	AC025174.1	1	INTRON	509	3.75623	PEL	13661
chr5	117569606	rs1479219	LINC02147	1	INTRON	46444	3.37708	PEL	6639
chr5	159072451	rs10515793	AC008691.1	3	INTRON	92441	3.52617	MXL	14772
chr5	164490804	rs4571494	AC109466.1	2	INTRON	90310	3.35525	PEL	28493
chr5	167941990	rs7709842	RARS	12	INTRON	28540	3.54993	PEL	16347
chr5	168108856	rs12521041	SLIT3	32	INTRON	618930	3.35108	PEL	108102
chr6	21819640	rs2208882	CASC15	6	INTRON	154637	3.50628	MXL	68769
chr6	22012236	rs12203803	CASC15	3	INTRON	113285	3.94623	MXL	201895
chr6	33670406	rs7759668	UQCC2	1	INTRON	9098	3.68015	MXL	30561
chr6	33699882	rs4713670	IP6K3	2	INTRON	14800	3.56953	MXL	30561
chr6	51617581	rs17753015	PKHD1	57	INTRON	334827	3.5784	MXL	19691
chr6	51894955	rs9370092	PKHD1	29	INTRON	57453	3.50579	PEL	9031
chr6	51896569	rs2435315	PKHD1	29	INTRON	55839	3.50579	PEL	9031
chr6	129432235	rs7738316	LAMA2	4	INTRON	227893	3.50753	MXL	9863
chr6	158304083	rs7741141	SNX9	4	INTRON	59787	3.47833	PEL	6025
chr6	158412832	rs10945973	SYNJ2	1	INTRON	9913	3.35056	PEL	8073
chr6	159172854	rs3123094	SYTL3	11	INTRON	90503	3.3242	PEL	8638
chr6	159225284	rs3127194	EZR	1	INTRON	13975	3.55612	PEL	8638
chr7	11272690	rs2107379	AC004160.1	1	INTRON	35756	3.71813	PEL	8575

chr7	38500353	rs2392577	AMPH	11	INTRON	170667	3.80947	PEL	52771
chr7	38500421	rs2392578	AMPH	11	INTRON	170599	4.70202	PEL	52771
chr7	38551053	rs10249892	AMPH	2	INTRON	119967	3.42387	MXL	709029
chr7	38551053	rs10249892	AMPH	2	INTRON	119967	3.78347	PEL	NA
chr7	42072483	rs846334	GLI3	7	INTRON	204129	3.30612	PEL	13180
chr7	103781481	rs194856	ORC5	12	INTRON	67014	3.83665	MXL	42464
chr7	111199127	rs4591961	IMMP2L	2	INTRON	3446	4.3777	CLM	16266
chr7	131995999	rs3906454	PLXNA4	3	INTRON	265324	3.43946	PEL	11844
chr7	133328222	rs10235846	EXOC4	10	INTRON	390393	3.68019	PEL	11052
chr7	133340630	rs2345943	EXOC4	10	INTRON	402801	3.32348	PEL	11052
chr7	133345635	rs7776986	EXOC4	10	INTRON	407806	3.30648	PEL	11052
chr7	133585386	rs6971638	EXOC4	12	INTRON	647557	3.59095	PEL	10900
chr7	133589608	rs6956135	EXOC4	12	INTRON	651779	3.59095	PEL	10900
chr7	136426247	rs7796504	AC009264.1	3	INTRON	422773	3.98583	PEL	9398
chr7	138617682	rs17160621	KIAA1549	1	INTRON	48382	3.58448	PEL	5098
chr7	142558133	rs8177123	EPHB6	1	INTRON	5267	4.66453	CLM	7327
chr7	142581288	rs4987613	AC245427.1	1	INTRON	7699	4.23981	PUR	12622
chr7	142581288	rs4987613	AC245427.1	1	INTRON	7699	4.25124	CLM	7327
chr7	142617214	rs4252461	TRPV5	8	INTRON	13691	4.07119	CLM	7327
chr8	4625732	rs10503276	CSMD1	1	INTRON	226491	3.3141	PEL	15739
chr8	9460449	rs4841179	TNKS	2	INTRON	47025	4.22429	PUR	30137
chr8	9462583	rs7832096	TNKS	2	INTRON	49159	4.15546	PUR	30137
chr8	9471060	rs4240626	TNKS	2	INTRON	57636	4.15546	PUR	30137
chr8	9571621	rs7461939	TNKS	11	INTRON	158197	3.86031	CLM	27121
chr8	94600795	rs4430060	LINC00535	5	INTRON	111866	3.43416	PEL	95210
chr8	108472213	rs16876325	ANGPT1	1	INTRON	38070	3.73527	PEL	53916
chr8	108492240	rs1433171	ANGPT1	1	INTRON	18043	3.70233	PEL	68048
chr9	71762766	rs10781440	TJP2	1	INTRON	26380	3.54517	MXL	20253
chr9	126355960	rs10986067	DENND1A	10	INTRON	336471	3.52364	MXL	16120
chr10	20428197	rs4237353	PLXDC2	3	INTRON	95948	3.33044	PEL	NA
chr10	21140255	rs703088	NEBL	10	INTRON	46276	3.53426	MXL	91411
chr10	59742330	rs2939583	MRPS35P3	1	EXON	285	3.40877	MXL	6936
chr10	64412122	rs2393908	AC024598.1	5	INTRON	278171	4.8568	PEL	9639
chr10	65626259	rs1916437	AC022387.1	2	INTRON	201835	3.44572	PEL	5876
chr10	68909644	rs10762143	CTNNA3	7	INTRON	546283	4.1173	PEL	12260
chr10	70960448	rs7893492	SUPV3L1	11	INTRON	20460	3.53846	PEL	10102
chr11	10669540	rs10840455	MRVI1	2	INTRON	45759	3.41158	MXL	73155
chr11	83123304	rs2510475	AP000446.1	3	INTRON	126133	3.82585	PEL	10558
chr12	1483235	rs12425175	ERC1	15	INTRON	382831	3.45477	PEL	3161
chr12	1483296	rs11061721	ERC1	15	INTRON	382892	3.45477	PEL	3161
chr12	88953326	rs3782179	KITLG	1	INTRON	20912	4.64881	BR	15484
chr12	88953326	rs3782179	KITLG	1	INTRON	20912	4.78707	PUR	12182
chr12	88953326	rs3782179	KITLG	1	INTRON	20912	3.49113	PEL	5118
chr12	88956400	rs11104952	KITLG	1	INTRON	17838	4.88227	BR	15484
chr12	88956400	rs11104952	KITLG	1	INTRON	17838	4.78675	PUR	12182
chr12	88956400	rs11104952	KITLG	1	INTRON	17838	3.49113	PEL	5118
chr12	108872988	rs4964714	LINC01498	3	INTRON	13374	3.73314	PEL	8851
chr12	114399157	rs12425509	RBM19	2	INTRON	5019	3.6908	PEL	21598
chr12	117423264	rs7131746	AC127164.1	1	INTRON	1878	3.8611	CLM	5042
chr12	117423264	rs7131746	AC127164.1	1	INTRON	1878	3.88194	MXL	5042
chr12	126895508	rs10744253	AC007368.1	9	INTRON	219811	3.46532	MXL	6162
chr12	126905611	rs11613705	AC007368.1	9	INTRON	229914	4.03659	MXL	6171
chr13	39682032	rs2324129	AL354809.1	1	INTRON	54823	3.40691	PEL	12096
chr13	39787406	rs7324440	AL354809.1	2	INTRON	160197	3.31384	PEL	7058
chr13	101002920	rs1888839	PCCA	18	INTRON	261651	3.4116	MXL	47287
chr13	114495335	rs7491764	TMEM255B	3	INTRON	24226	4.26395	CLM	NA
chr13	114495335	rs7491764	TMEM255B	3	INTRON	24226	5.35662	PUR	NA
chr13	114495335	rs7491764	TMEM255B	3	INTRON	24226	4.58873	MXL	NA
chr14	25284005	rs898766	STXBP6	5	INTRON	235498	4.24069	MXL	10358
chr14	25284005	rs898766	STXBP6	5	INTRON	235498	3.41304	PEL	6808
chr14	27179913	rs12435522	LINC02588	1	INTRON	73466	3.53235	MXL	5940
chr14	69543224	rs10145565	DCAF5	6	INTRON	76619	3.47568	PEL	10305
chr15	36327528	rs4502172	AC021351.1	2	INTRON	216930	3.87935	PEL	3916
chr15	40059535	rs12440567	FSIP1	3	INTRON	15496	3.81376	PEL	NA
chr15	43039063	rs7175582	TTBK2	14	INTRON	173614	3.38804	PEL	18700
chr15	45050593	rs2290331	TRIM69	3	INTRON	22033	4.4346	PUR	9358
chr15	57894443	rs7178159	GCOM1	1	INTRON	10212	3.80653	MXL	58566
chr15	58246768	rs3204690	ALDH1A2	13	EXON	111848	3.60967	PEL	10605
chr15	78334655	rs11634607	TBC1D2B	3	INTRON	35339	3.76965	PEL	8661
chr15	100974315	rs1966764	CERS3	12	INTRON	110610	3.36747	PEL	12239
chr16	11193930	rs3893660	CLEC16A	18	INTRON	155492	3.80614	PEL	5595
chr16	63093010	rs7203144	AC040174.1	4	INTRON	70548	3.50044	MXL	16302
chr16	72694352	rs1429433	LINC01572	1	INTRON	4551	3.83997	PEL	7941
chr16	78524099	rs2738710	WWOX	4	INTRON	390548	3.38544	MXL	8679
chr16	78524099	rs2738710	WWOX	4	INTRON	390548	4.22091	CLM	7537
chr17	55437411	rs703859	MSI2	5	INTRON	103516	3.39257	MXL	12985
chr18	11765356	rs8094039	GNAL	5	INTRON	13849	3.57292	MXL	5047
chr18	11774500	rs2161961	GNAL	5	INTRON	22993	3.55793	MXL	5047
chr18	29797921	rs616114	GAREM1	1	INTRON	69137	3.36921	PEL	12267
chr18	50622857	rs9957443	DCC	6	INTRON	344325	3.447	PEL	8928
chr18	53727502	rs2535724	AC006305.1	3	INTRON	130991	3.90342	CLM	251231
chr18	55355822	rs17759405	AC027097.2	2	INTRON	49135	3.54755	MXL	56547
chr19	33581219	rs12608825	GPATCH1	2	INTRON	9433	3.43519	PEL	17443

chr20	34190870	rs3746410	FER1L4	6	INTRON	2378	3.39706	MXL	10278
chr20	42882209	rs6031477	GDAP1L1	1	INTRON	6322	3.72565	PEL	21411
chr20	52567546	rs11086446	BCAS1	9	INTRON	119538	3.66912	PEL	19877
chr21	38306954	rs2835537	HLCS	5	INTRON	55582	3.44629	MXL	6745
chr21	38323528	rs2835545	HLCS	2	INTRON	39008	3.44629	MXL	6745
chr21	41161654	rs760285	IGSF5	6	INTRON	44320	4.00826	MXL	7914
chr21	41161654	rs760285	IGSF5	6	INTRON	44320	3.85064	PEL	7894
chr22	19483898	rs2073759	CDC45	7	INTRON	16549	4.02527	CLM	34320
chr22	28833502	rs695388	TTC28	2	INTRON	242351	3.30197	PEL	70060
chr22	49123661	rs6587310	FAM19A5	3	INTRON	238257	3.32571	PEL	17822
