# Supplementary material for: Demographic history differences between Hispanics and Brazilians imprint haplotype features
Source: G3 (Bethesda). 2022 May 2;12(7):jkac111. doi: 10.1093/g3journal/jkac111 (PMC9258545; doi:10.1093/g3journal/jkac111)
Supplement: jkac111_Supplemental_Table_S4 [file jkac111_supplemental_table_s4.pdf]

Supplementary Table S4. Intergenic markers identified in putatuvely positive selection using iHS. TSS: transcription starting site, TMRCA: time to most recent common ancestor. YBP: years before present.

Chromosome	Position	rsID	Nearest Gene	Area	Distance	TSSDistance	Normalized iHS	Population	TMRCA (YBP)
chr1	64238724	rs589465	ROR1	PROMOTER	969	-969	4.07285	MXL	4460
chr1	64778399	rs1496023	UBE2U	DOWNSTREAM	45348	109089	3.40358	MXL	41662
chr1	89549958	rs10801713	GBP1	UPSTREAM	18915	-18915	3.31687	PEL	14280
chr1	111925349	rs4839130	PGCP1	DOWNSTREAM	133	9092	3.49204	PEL	7672
chr1	119552284	rs4501872	AL139420.2	UPSTREAM	8256	-8256	3.40327	PEL	10952
chr1	153468701	rs16835368	RN7SL44P	DOWNSTREAM	4242	-4242	3.79905	MXL	4991
chr1	172696653	rs859648	SLC25A38P1	UPSTREAM	21047	-21047	3.47361	PEL	9292
chr1	180113196	rs10753206	QSOX1	UPSTREAM	10773	-10773	4.05426	MXL	5435
chr1	180113196	rs10753206	QSOX1	UPSTREAM	10773	-10773	4.00509	PEL	4726
chr2	2372168	rs6759202	MYT1L	UPSTREAM	36732	-36732	3.35087	PEL	14416
chr2	25413309	rs4665287	LINC01381	UPSTREAM	13873	-13873	3.43386	PEL	7233
chr2	41733169	rs4985357	AC010739.1	UPSTREAM	90357	-90357	3.70294	PEL	13819
chr2	41746823	rs6726218	AC010739.1	UPSTREAM	76703	-76703	3.70294	PEL	13819
chr2	82035216	rs10166510	AC013262.1	UPSTREAM	141364	141615	3.82999	CLM	291361
chr2	82035872	rs10190986	AC013262.1	UPSTREAM	142020	142271	3.91239	CLM	352962
chr2	103019919	rs1035127	IL18R1	DOWNSTREAM	4684	47176	3.57586	PEL	17280
chr2	169306579	rs2724184	RN7SL813P	PROMOTER	1255	-1255	3.38887	MXL	86018
chr2	178462914	rs1548067	AC073834.1	UPSTREAM	4903	-4903	4.2897	CLM	11404
chr2	178462914	rs1548067	AC073834.1	UPSTREAM	4903	-4903	3.80587	MXL	11404
chr2	178462914	rs1548067	AC073834.1	UPSTREAM	4903	-4903	3.30855	PEL	9381
chr2	223873700	rs10200334	KCNE4	UPSTREAM	42832	-42832	3.91162	CLM	9128
chr2	223873700	rs10200334	KCNE4	UPSTREAM	42832	-42832	3.52856	MXL	9085
chr2	223900504	rs1155818	KCNE4	UPSTREAM	16028	-16028	3.49007	MXL	9066
chr3	95225762	rs7631830	MTHFD2P1	DOWNSTREAM	147505	176275	3.51587	MXL	27968
chr4	12539448	rs6822224	ECM1P2	UPSTREAM	101262	-101262	4.05567	MXL	22978
chr4	38745482	rs10008032	RNA5SP158	DOWNSTREAM	14930	-14930	3.42017	PEL	49153
chr4	60018418	rs357814	AC108517.1	TSS	134	-134	3.44709	MXL	58662
chr4	67875548	rs6831786	RNU6-699P	DOWNSTREAM	112459	112568	3.86348	CLM	60139
chr4	102283898	rs10433982	FLJ20021	DOWNSTREAM	13858	14961	3.31556	PEL	30873
chr4	158390219	rs9784453	GRIA2	DOWNSTREAM	102992	248349	3.59171	MXL	4004
chr4	170230419	rs12511773	SH3RF1	UPSTREAM	38163	-38163	4.5398	MXL	5588
chr4	170230625	rs13115031	SH3RF1	UPSTREAM	38369	-38369	3.82953	MXL	5588
chr4	170248489	rs4692714	SH3RF1	UPSTREAM	56233	-56233	3.9751	MXL	5624
chr5	38009611	rs11955767	LINC02107	UPSTREAM	16188	-16188	3.42193	MXL	6965
chr5	38010364	rs1567327	LINC02107	UPSTREAM	15435	-15435	3.51994	MXL	6965
chr5	73609473	rs16871648	RN7SL814P	UPSTREAM	4338	-4338	3.4298	PEL	62692
chr5	121101384	rs11955857	FTMT	UPSTREAM	86266	-86266	3.96049	CLM	12669
chr5	154853855	rs1295240	AC008725.1	UPSTREAM	17041	-17041	3.51895	PEL	10333
chr5	154863737	rs1484277	AC008725.1	UPSTREAM	7159	-7159	3.52094	PEL	10333
chr5	154942606	rs13355365	AC008725.1	DOWNSTREAM	69448	71710	3.5911	PEL	10333
chr5	154942791	rs2312974	AC008725.1	DOWNSTREAM	69633	71895	3.58773	PEL	10333
chr5	160280340	rs4921346	ATP10B	PROMOTER	1119	-1119	3.53958	PEL	70502
chr6	51175375	rs17813135	AL158050.2	DOWNSTREAM	74705	-74705	3.29854	PEL	44997
chr6	52534078	rs7762854	AL109918.1	DOWNSTREAM	125	4850	3.54724	PEL	7949
chr6	53107320	rs9463885	HMGB1P20	UPSTREAM	6225	6901	3.85159	PEL	60234
chr6	78678372	rs16889550	MEI4	DOWNSTREAM	41627	318381	3.55602	MXL	281653
chr6	127061318	rs853969	RPS4XP9	DOWNSTREAM	56326	57137	3.76981	PEL	15249
chr7	103706936	rs4727586	ORC5	DOWNSTREAM	59852	141559	3.64135	MXL	58363
chr8	4988269	rs11778306	AC019176.2	UPSTREAM	2514	2828	3.41724	PEL	7887
chr8	32753916	rs12548196	RNU6-663P	DOWNSTREAM	15095	-15095	3.82206	PEL	109447
chr8	59229893	rs7006132	AC092819.1	UPSTREAM	45215	-45215	3.48578	PEL	15745
chr8	59307275	rs897735	UBXN2B	UPSTREAM	16548	-16548	3.66047	PEL	12362
chr8	76733437	rs7828571	AC016194.1	UPSTREAM	34742	34837	3.78607	MXL	179973
chr8	115193169	rs2048795	AC064802.1	UPSTREAM	101127	-101127	3.60074	MXL	31388
chr9	11359686	rs1335445	AL451129.1	UPSTREAM	83372	-83372	3.39507	MXL	61904
chr9	13961462	rs7856131	LINC00583	DOWNSTREAM	15853	33492	3.32952	PEL	6372
chr9	14429307	rs4741377	NFIB	UPSTREAM	30325	-30325	3.44775	MXL	12415
chr9	94294473	rs16907574	ROR2	DOWNSTREAM	30900	416689	3.53695	MXL	13397
chr9	94294473	rs16907574	ROR2	DOWNSTREAM	30900	416689	3.77323	PEL	7084
chr10	4149756	rs10751958	AC025822.2	DOWNSTREAM	18551	55838	3.40085	PEL	5567
chr10	36090523	rs10764058	PCAT5	DOWNSTREAM	675	23293	4.15204	PUR	23775
chr10	36090652	rs977677	PCAT5	DOWNSTREAM	804	23422	4.19537	MXL	18140
chr10	59744524	rs1930455	MRPS35P3	DOWNSTREAM	1771	2479	3.40877	MXL	6936
chr10	59744545	rs1930456	MRPS35P3	DOWNSTREAM	1792	2500	3.40877	MXL	6936
chr10	70877228	rs10998581	VPS26A	UPSTREAM	6040	-6040	3.50638	PEL	10109
chr10	115285900	rs11196359	HABP2	UPSTREAM	24696	-24696	3.39052	PEL	10057
chr10	125090399	rs11248496	AL160290.3	UPSTREAM	7297	7461	3.32766	PEL	37299
chr11	25432895	rs12277768	AC015820.1	UPSTREAM	125929	-125929	4.06633	PEL	7075
chr11	25528202	rs10767396	AC015820.1	UPSTREAM	30622	-30622	3.37516	PEL	7075
chr11	25540483	rs10501011	AC015820.1	UPSTREAM	18341	-18341	4.17339	PEL	7075
chr11	25550582	rs11028752	AC015820.1	UPSTREAM	8242	-8242	4.17339	PEL	7075
chr11	30261716	rs1340034	FSHB	DOWNSTREAM	4908	9153	4.04381	MXL	330448
chr11	98251961	rs10501885	AP003038.1	DOWNSTREAM	183843	-183843	3.76669	MXL	132960
chr11	106047102	rs4505037	AP001001.2	UPSTREAM	44259	-44259	3.51304	MXL	14488
chr11	123813057	rs4935852	OR6T1	DOWNSTREAM	435	-435	3.92524	CLM	181776
chr12	108778192	rs720051	CMKLR1	UPSTREAM	45074	-45074	3.84858	PEL	9595
chr12	108778441	rs4964689	CMKLR1	UPSTREAM	45323	-45323	3.84858	PEL	9595
chr12	126238948	rs4765066	AC005252.2	UPSTREAM	54266	-54266	3.47234	PEL	10864
chr13	89063181	rs380603	AL355677.1	DOWNSTREAM	61225	66341	3.7881	PEL	13139
chr13	89071581	rs424128	AL355677.1	DOWNSTREAM	52825	57941	3.7881	PEL	13139
chr13	90623215	rs9588815	PEX12P1	UPSTREAM	20870	-20870	3.41982	MXL	13689
chr13	104199575	rs9514153	ATP6V1G1P7	UPSTREAM	102257	102605	3.43925	MXL	13003
chr13	104268943	rs1360609	ATP6V1G1P7	UPSTREAM	171625	171973	4.36176	PUR	14547
chr13	104268943	rs1360609	ATP6V1G1P7	UPSTREAM	171625	171973	3.74421	PEL	6823
chr13	104268943	rs1360609	ATP6V1G1P7	UPSTREAM	171625	171973	3.51533	MXL	13003
chr13	105153074	rs9300935	RPL7P45	UPSTREAM	313604	-313604	3.39056	MXL	9121
chr14	48151247	rs8015943	MDGA2	UPSTREAM	7090	-7090	3.63645	PEL	7937
chr14	55308004	rs17739146	GCH1	DOWNSTREAM	722	61538	3.36188	PEL	66156

chr15	58245151	rs4646645	ALDH1A2	DOWNSTREAM	471	112970	3.73158	PEL	10605
chr15	68109907	rs28670272	SKOR1	UPSTREAM	2135	-2135	4.12896	MXL	35875
chr16	79513098	rs11150167	AC084064.1	UPSTREAM	26402	-26402	3.4833	MXL	26306
chr17	50309871	rs12949235	snoZ178	UPSTREAM	6164	6253	4.40216	CLM	264600
chr17	69926024	rs9905128	AC005144.1	UPSTREAM	58133	-58133	3.3917	MXL	37597
chr18	58676641	rs7233376	AC113137.1	UPSTREAM	106682	-106682	3.7861	PEL	120535
chr18	68654632	rs11151626	AC090415.2	DOWNSTREAM	13081	24094	3.45655	MXL	12979
chr20	12661944	rs6041542	LINC01722	DOWNSTREAM	183906	271223	3.29462	PEL	7377
chr20	12666256	rs6078720	LINC01722	DOWNSTREAM	179594	266911	3.52848	PEL	7377
chr20	44311354	rs4810465	WFDC10B	DOWNSTREAM	1938	22304	3.84941	PEL	15146
chr21	17069166	rs2823455	RAD23BP3	PROMOTER	266	-266	3.81976	MXL	6778
chr22	44769667	rs4823354	Z85994.1	DOWNSTREAM	7503	8236	3.31273	PEL	3260
